# PBX1 Improves Cognition and Reduces Amyloid‐β Pathology in APP/PS1 Mice by Transcriptionally Activating the CRTC2–CREB Pathway

**DOI:** 10.1111/acel.70311

**Published:** 2025-12-04

**Authors:** Zinan Liu, Xiangyuan Meng, Rifeng Lu, Xiaoting Meng, Siyao Li, Yujie Wang, Xinpeng Liu, Xiaomei Liu, Jinyu Liu

**Affiliations:** ^1^ Department of Toxicology, School of Public Health Jilin University Changchun China; ^2^ Department of Histology & Embryology, College of Basic Medical Sciences Jilin University Changchun China

**Keywords:** Alzheimer's disease, amyloid β deposition, cognitive rescue, CRTC2/CREB, PBX1

## Abstract

Alzheimer's disease (AD) is characterized by progressive cognitive decline, amyloid β (Aβ) deposition, and synaptic dysfunction. However, the mechanisms underlying neurodegeneration remain poorly understood. In this study, we investigated the therapeutic potential of PBX1, a transcriptional regulator implicated in neurodevelopment and neuroprotection, against AD. PBX1 expression was significantly downregulated in postmortem hippocampal tissues from patients with AD and in the APP/PS1 mouse model. In vitro, PBX1a knockdown reduced neurite complexity and increased apoptosis. PBX1a overexpression reversed these effects and reduced soluble Aβ_1–40_ and Aβ_1–42_ levels. In vivo, hippocampal overexpression of PBX1a restored spatial learning and memory, reduced Aβ burden by 41%, and increased neurite length by 1.5‐fold. These behavioral and structural improvements were accompanied by reduced levels of hyperphosphorylated Tau and toxic Aβ oligomers. Mechanistically, PBX1 directly activated the transcription of CRTC2—a coactivator of CREB, thereby increasing CRTC2 expression and its nuclear colocalization with phosphorylated CREB. Restoration of the PBX1–CRTC2–CREB axis enhanced neuronal survival and synaptic integrity. Notably, CRTC2 knockdown blocked PBX1‐mediated reductions in Aβ deposition, apoptosis, and hyperphosphorylated Tau expression, confirming the role of the PBX1–CRTC2–CREB axis in conferring neuroprotection. Together, our findings indicate that PBX1 is a key modulator of neuronal resilience in AD and that it functions through transcriptional activation of the CRTC2/CREB pathway. By unraveling a mechanism that links transcriptional regulation to amyloid clearance and cognitive function, this study highlights PBX1 as a promising therapeutic target for AD.

## Graphical abstract

We found that the transcription factor PBX1 is markedly downregulated in patients with Alzheimer's disease and in disease models. Restoring PBX1 reduces Aβ pathology and rescues cognitive deficits. Mechanistically, PBX1 directly activates the transcription of CRTC2 and enhances p‐CREB signaling, thereby conferring neuroprotection.

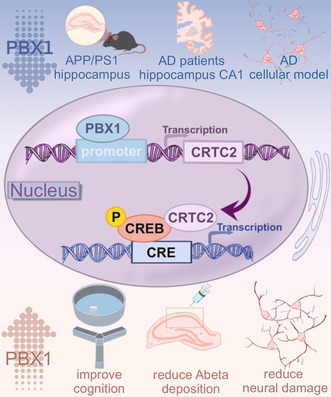

**Abbreviations**
ad
: Alzheimer's disease
APP/PS1: APP Swedish mutation (APPswe)/PSEN1dE9
APPswe: Swedish mutation K670N/M671L
Aβ: amyloid‐β
CREB: cAMP response element‐binding protein
CRTC2: CREB‐regulated transcription coactivator 2
GEO: Gene Expression Omnibus
GSK3β: glycogen synthase kinase‐3beta
hSyn: human synapsin promoter
MWM: Morris water maze
PBX1: pre‐B‐cell leukemia transcription factor 1
PD: Parkinson's disease
p‐TAU: phosphorylated Tau
Tuj1: βIII‐tubulin3

## 1 Introduction

Alzheimer's disease (AD) is a progressive age‐associated neurodegenerative disorder characterized by cognitive decline, memory impairment, and socioemotional disturbances. Its pathological hallmarks include amyloid β (Aβ) deposition and neurofibrillary tangles formed by hyperphosphorylated Tau protein aggregates (Paasila et al. 2021). The pathogenesis of AD remains poorly understood, and most therapies targeting the pathological mechanisms have repeatedly failed in clinical trials (Congdon et al. 2023; van Dyck et al. 2023; Whitehouse and Saini 2022; Zhang et al. 2023). Currently, few definitive disease‐modifying therapies are available for AD.

Researchers have increasingly explored the role of the transcriptional regulator pre‐B‐cell leukemia transcription factor 1 (PBX1) in neurodegenerative diseases. A multiomics integrative analysis revealed that PBXIP1, a corepressor of PBX1 (Abramovich et al. 2002, 2000; Manavathi et al. 2012), is aberrantly upregulated in the brain tissues of patients with AD (Zhang, Sun, et al. 2024). The analysis further suggested that PBXIP1 is strongly and positively correlated with aging and three key neuropathological features of AD—Aβ load, tangle density, and neurofibrillary tangle burden (Zhang, Sun, et al. 2024). Analysis of the AlzData database (http://www.alzdata.org/, CFG RANK module) indicated that PBX1 expression is strongly and negatively correlated with the severity of both the Aβ and the phosphorylated Tau (p‐Tau) pathological burden in AD mouse models (Xu et al. 2018). In human brain samples, PBX1 shows only a modest decrease in bulk transcriptomic datasets (Johnson et al. 2022), but single‐nucleus multi‐omic profiling of late‐stage AD reveals a more pronounced reduction of PBX1 within specific excitatory neuron subpopulations (Morabito et al. 2021), consistent with its association with Aβ and p‐Tau burden in model systems. As a transcription factor with a three‐amino‐acid‐loop‐extension homeodomain, PBX1 regulates gene expression in a spatiotemporally controlled manner (DiMartino et al. 2001; Ficara et al. 2008; Gordon et al. 2010; Jiang et al. 2019; Zhou et al. 2020). In the central nervous system, PBX1 regulates neurodevelopment and exerts neuroprotective effects. Furthermore, PBX1 maintains hippocampal neurogenesis through the protein kinase B/glycogen synthase kinase 3β (GSK3β)/cyclic adenosine monophosphate response element‐binding protein (CREB) pathway (Wang et al. 2024) and acts as a neurogenic pioneer factor in the subventricular zone, promoting the differentiation of neural progenitor cells into neurons and supporting the survival of newly generated neurons (Grebbin et al. 2016; Grebbin and Schulte 2017; Remesal et al. 2020). In Parkinson's disease (PD)—another neurodegenerative disorder—PBX1 expression is markedly downregulated concomitant with dopaminergic neuron loss (Villaescusa et al. 2016). Restoration of PBX1 expression in cellular models of PD was demonstrated to reduce apoptosis (Li et al. 2022).

On the basis of the aforementioned findings, we hypothesized that PBX1 would play a role in AD pathogenesis. In this study, we investigated changes in PBX1 levels in cellular and mouse models of AD. Our findings revealed that PBX1 expression was downregulated in hippocampal tissues from both patients with AD and APP/PS1 mice. PBX1 overexpression mitigated cognitive deficits and reduced Aβ deposition. Furthermore, PBX1 bound directly to the promoter region of the CREB‐regulated transcription coactivator 2 gene (*CRTC2*), activating the transcription of *CRTC2* and enhancing the transcriptional activity of CREB, thereby exerting neuroprotective effects. These findings highlight PBX1 as a potential therapeutic target for AD.

## 2 Results

### 2.1 PBX1 Expression Is Downregulated in the Hippocampal Tissues of Both Patients With AD and APP/PS1 Mice

**FIGURE 1:**
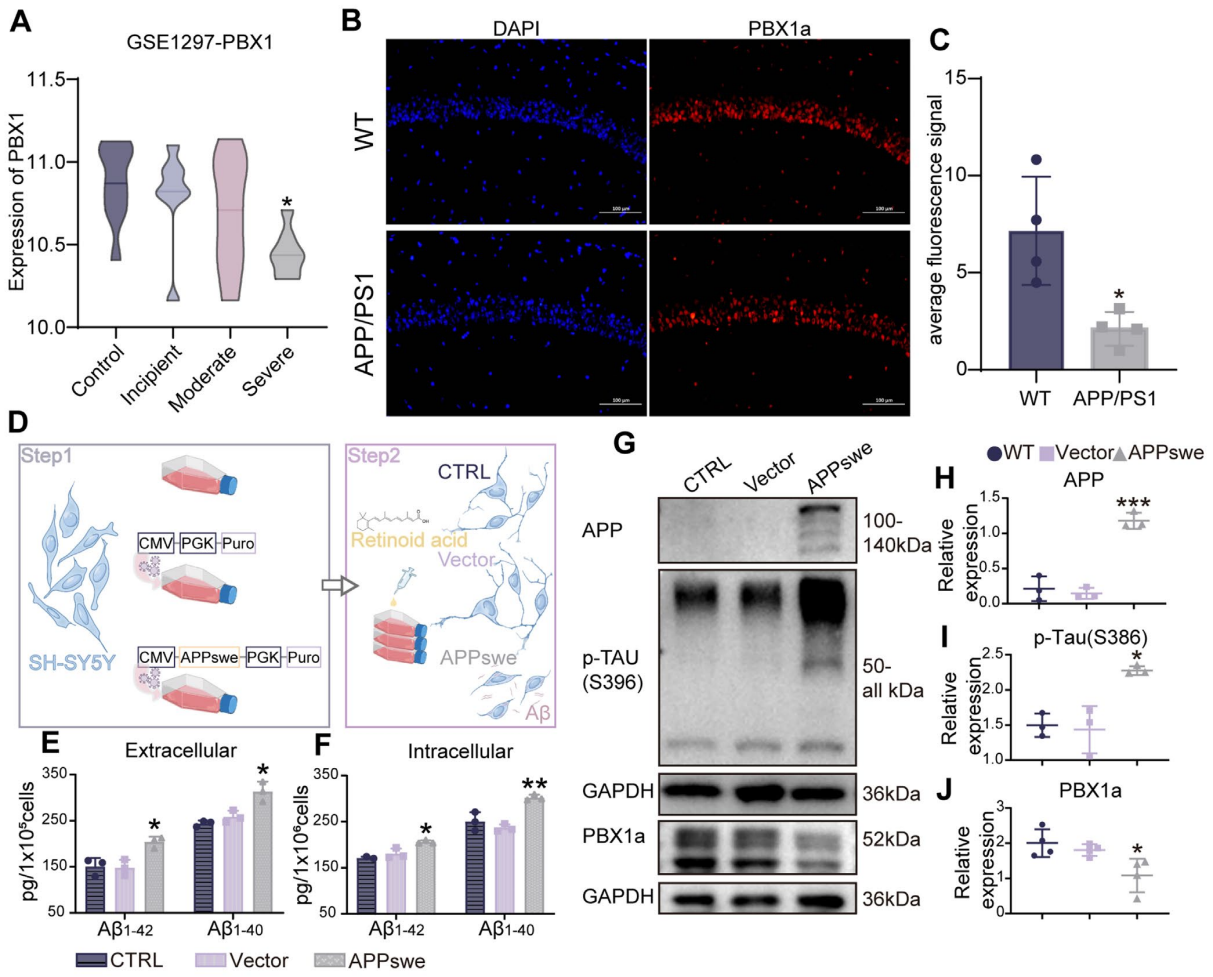
PBX1 expression was downregulated in patients with AD and in animal and cellular models of AD. (A) PBX1 expression was downregulated in the hippocampal CA1 neurons of patients with varying levels of AD severity (*n* = 22; stratified by disease severity as follows: Incipient AD, *n* = 7; moderate AD, *n* = 8; and severe AD, *n* = 9) compared with the expression levels in individuals with normal cognitive function (*n* = 9). Data were collected from the GEO dataset GSE1297. Kruskal–Wallis tests were performed followed by Dunn's post hoc pairwise comparisons with Holm p‐value correction. (B and C) Immunofluorescence of PBX1 from the hippocampal CA1 neurons of wild‐type and APP/PS1 mice. Each experimental group comprised four mice; the independent‐samples *t*‐test was performed. (D) Cellular model of AD. APPswe‐overexpressing SH‐SY5Y cells were treated with retinoic acid. (E and F) Levels of intracellular and extracellular Aβ_1–42_ and Aβ_1–40_, measured through an enzyme‐linked immunosorbent assay. Statistical test: One‐way ANOVA with Tukey's multiple‐comparison test. (G) Western blot analysis of APP, p‐Tau(S396), and PBX1 expression following APPswe overexpression. (H–J) Scatter plots depicting the expression levels of APP, p‐Tau(S396), and PBX1 normalized to that of GAPDH. Statistical test: One‐way ANOVA with Tukey's multiple‐comparison test. *p < 0.05, **p < 0.01, and ***p < 0.001.

Analysis of data from the Gene Expression Omnibus (GEO) database revealed that PBX1 expression was significantly downregulated in AD (*p* = 0.03; Figure 1A). Given the mature neuron‐specific expression of PBX1a, an isoform of PBX1, in the brain (Remesal et al. 2020), we focused on PBX1a for subsequent analyses. We measured the expression level of PBX1a in APP/PS1 mice and found that it was significantly lower in the hippocampal CA1 neurons of transgenic mice than in those of wild‐type mice (*p* = 0.014; Figure 1B,C). SH‐SY5Y cells, which can differentiate into cholinergic marker‐expressing neuron‐like cells following retinoic acid treatment (Figure S1), were transduced with APP containing the Swedish mutation (APPswe) and were subsequently treated with retinoic acid to generate a cellular model of AD (Figure 1D). A vector control group was also established. Compared with this group, the APPswe group exhibited significantly increased levels of soluble Aβ_1–40_ and Aβ_1–42_ (extracellular: Aβ_1–42_ *p* = 0.01, Aβ_1–40_ *p* = 0.019, intracellular: Aβ_1–42_ *p* = 0.02, Aβ_1–40_ *p* = 0.001; Figure 1E,F), along with reduced PBX1 levels (Figure 1G–J). These findings indicate downregulation of PBX1 expression in AD.

### 2.2 Knockdown of PBX1 Impairs Neurite Outgrowth and Increases Neuronal Apoptosis

**FIGURE 2:**
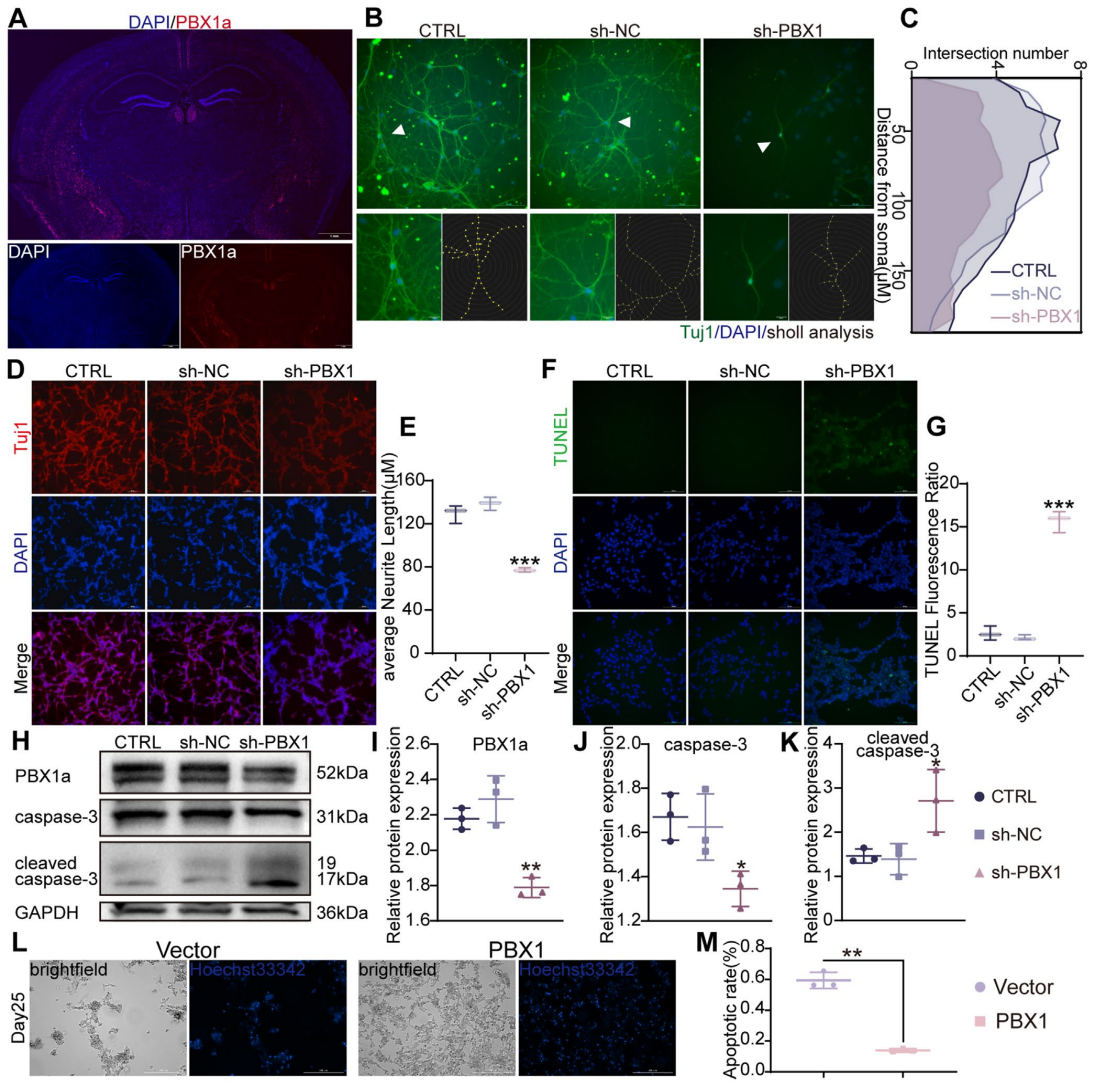
PBX1 knockdown reduced neurite complexity and increased apoptosis. (A) PBX1a expression in the hippocampus, cortex, and striatum of wild‐type C57BL/6J mice (*n* = 3). (B and C) Immunofluorescence of Tuj1 and neurite branching of primary hippocampal neurons transduced with either sh‐NC (negative control) or sh‐PBX1 lentiviral particles. Each experimental group comprised 11 to 12 neurons. (D and E) Immunofluorescence of Tuj1 and neurite length of differentiated SH‐SY5Y cells transduced with either sh‐NC or sh‐PBX1 lentiviral particles. (F and G) TUNEL staining results and a histogram depicting the staining intensity normalized to the area of DAPI‐positive differentiated SH‐SY5Y cells. (H–K) Levels of caspase‐3 and cleaved caspase‐3 following PBX1 knockdown, measured through Western blotting. The scatter plots depict the expression levels of these proteins normalized to that of GAPDH. (L and M) Hoechst 33342 staining of differentiated SH‐SY5Y cells cultured in B‐27 and subjected to an apoptotic assay. Statistical analyses were performed using one‐way analysis of variance with Tukey's multiple comparison test unless otherwise specified. *p < 0.05, **p < 0.01, and ***p < 0.001.

To evaluate the neuroprotective potential of PBX1 against AD, we first evaluated the expression pattern of this protein. Most studies have evaluated PBX1 expression in midbrain dopaminergic neurons (Grebbin et al. 2016; Sgadò et al. 2012; Villaescusa et al. 2016), highlighting its vital roles in the differentiation, maturation, and survival of neurons. However, the activity of PBX1 in the hippocampus, a region central to AD pathogenesis, remains unclear. Therefore, we subjected the brain tissues of wild‐type mice to immunofluorescence staining. PBX1a was detected in the mouse cerebral cortex, hippocampus and hypothalamus (Figure 2A). Quantitative analysis across hippocampal subregions (CA1, CA3 and DG) showed no significant differences in PBX1a expression (*p* > 0.05; Figure S2). Notably, knocking down PBX1a in primary hippocampal cells significantly reduced neurite complexity (*p* = 0.013; Figure 2B,C) and upregulated cleaved caspase (a biomarker of apoptosis) expression (Figure S3). However, PBX1a knockdown unexpectedly had no significant effect on the expression of neuronal markers (Tau, NeuN, Tuj1, and NSE) in SH‐SY5Y‐derived neuron‐like cells, as indicated by Western blotting (Figure S4). Nonetheless, immunofluorescence staining revealed reduced neurite outgrowth (*p* < 0.001; Figure 2D,E), accompanied by increased staining intensity in the terminal deoxynucleotidyl transferase deoxyuridine triphosphate nick end labeling (TUNEL) assay and elevated cleaved caspase‐3 levels, indicating increased apoptosis (Figure 2F–K). Conversely, overexpression of PBX1a in SH‐SY5Y cells increased the neurite complexity of neuron‐like cells (Figure S5), ameliorated apoptosis, and extended the in vitro survival of differentiated cells (Figure 2L,M). Collectively, these findings indicate that PBX1a is essential for neurite outgrowth and neuronal survival.

### 2.3 PBX1 Overexpression Ameliorates Core AD Pathologies In Vitro

**FIGURE 3:**
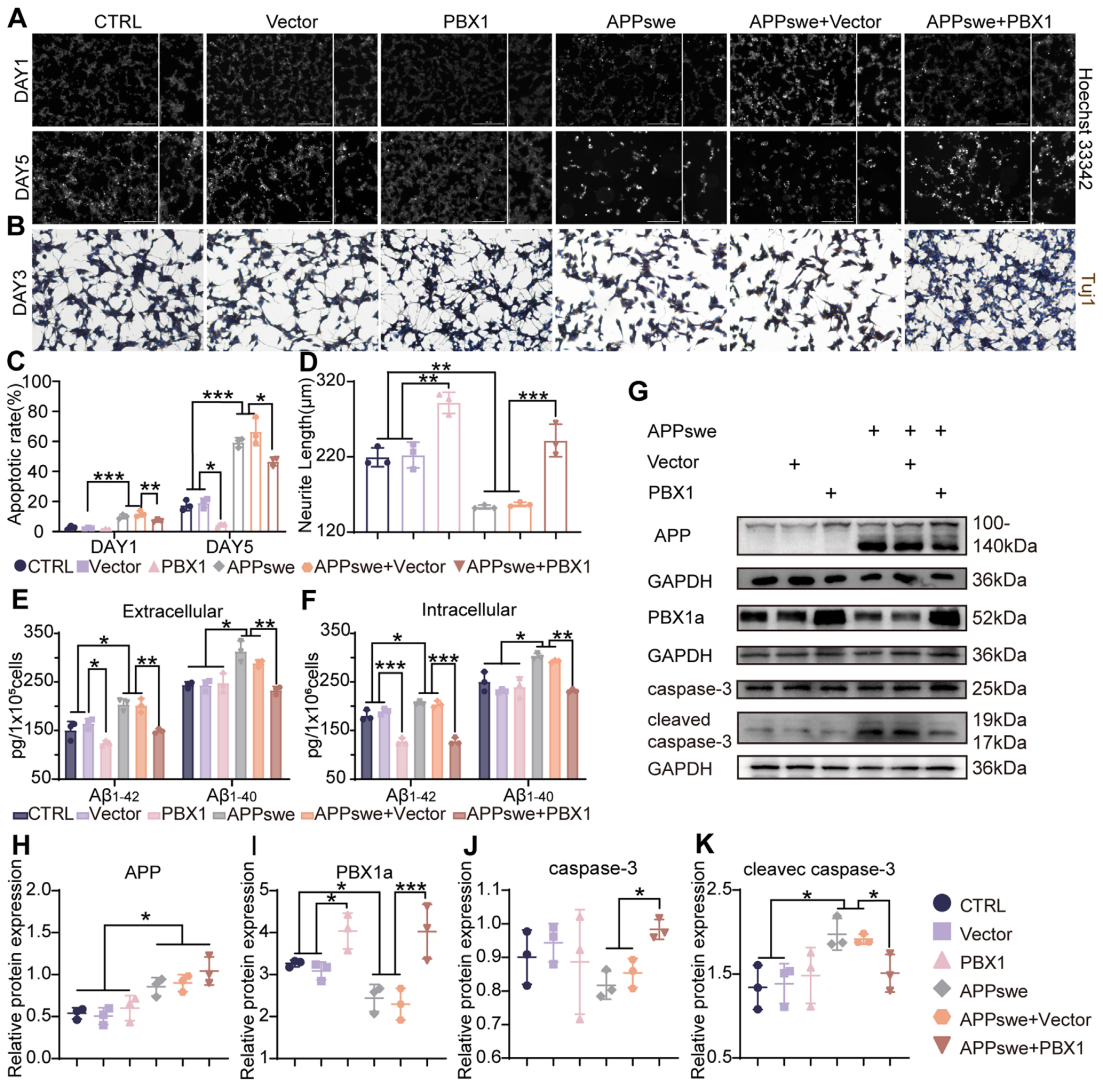
PBX1 overexpression attenuated neurite deficits, Aβ deposition, and apoptosis in a cellular model of AD. (A and C) Hoechst 33342 staining of differentiated SH‐SY5Y cells cultured in Neurobasal medium with B‐27 and subjected to an apoptotic assay. (B and D) Tuj1 immunocytochemistry was performed using Day 3 cell culture to measure neurite length. (E and F) Extracellular and intracellular levels of soluble Aβ_1–42_ and Aβ_1–40_ in APPswe/PBX1 cells, measured through an enzyme‐linked immunosorbent assay. (G) Levels of APP, PBX1a, caspase‐3, and cleaved caspase‐3 in APPswe/PBX1‐overexpressing cells, measured through Western blotting. (H–K) Expression levels of these proteins were normalized to that of GAPDH. Statistical analyses were performed using one‐way analysis of variance with Tukey's multiple comparison test unless otherwise specified. *p < 0.05, **p < 0.01, and ***p < 0.001.

Considering the neuroprotective role of PBX1 in basic neuronal models, we investigated its function in an in vitro AD model established by overexpressing APPswe in SH‐SY5Y cells. The model showed downregulation of PBX1, so we restored PBX1 expression by overexpression. PBX1‐overexpressing cells exhibited marked reductions in apoptosis. Hoechst 33342 staining (Days 1 and 5) revealed reduced apoptosis in APPswe+PBX1‐overexpressing cells compared with the findings in vector control cells (Figure 3A,C). This finding was accompanied by a lower level of cleaved caspase‐3 in APPswe+PBX1‐overexpressing cells than in vector control cells (Figure 3G–K). Tuj1 immunocytochemistry indicated reversal of neurite length deficits, with the neurites being significantly longer for PBX1‐overexpressing neurons than for vector control cells (241.7 ± 21.7 vs. 157.0 ± 3.0 μm; *p* < 0.001; Figure 3B,D). Furthermore, PBX1 overexpression significantly reduced Aβ burden, as indicated by an enzyme‐linked immunosorbent assay (ELISA): reductions were noted in both intracellular and extracellular levels of soluble Aβ_1–42_ and Aβ_1–40_ (APPswe+Vector vs. APPswe+PBX1; extracellular: Aβ_1–42_ *p* = 0.001, Aβ_1–40_ *p* = 0.004, intracellular: Aβ_1–42_ *p* < 0.001, Aβ_1–40_ *p* = 0.002; Figure 3E,F). Collectively, these findings suggest that restoration of PBX1 expression ameliorates core AD pathologies in vitro.

### 2.4 PBX1 Overexpression Improves Memory in APP/PS1 Mice

**FIGURE 4:**
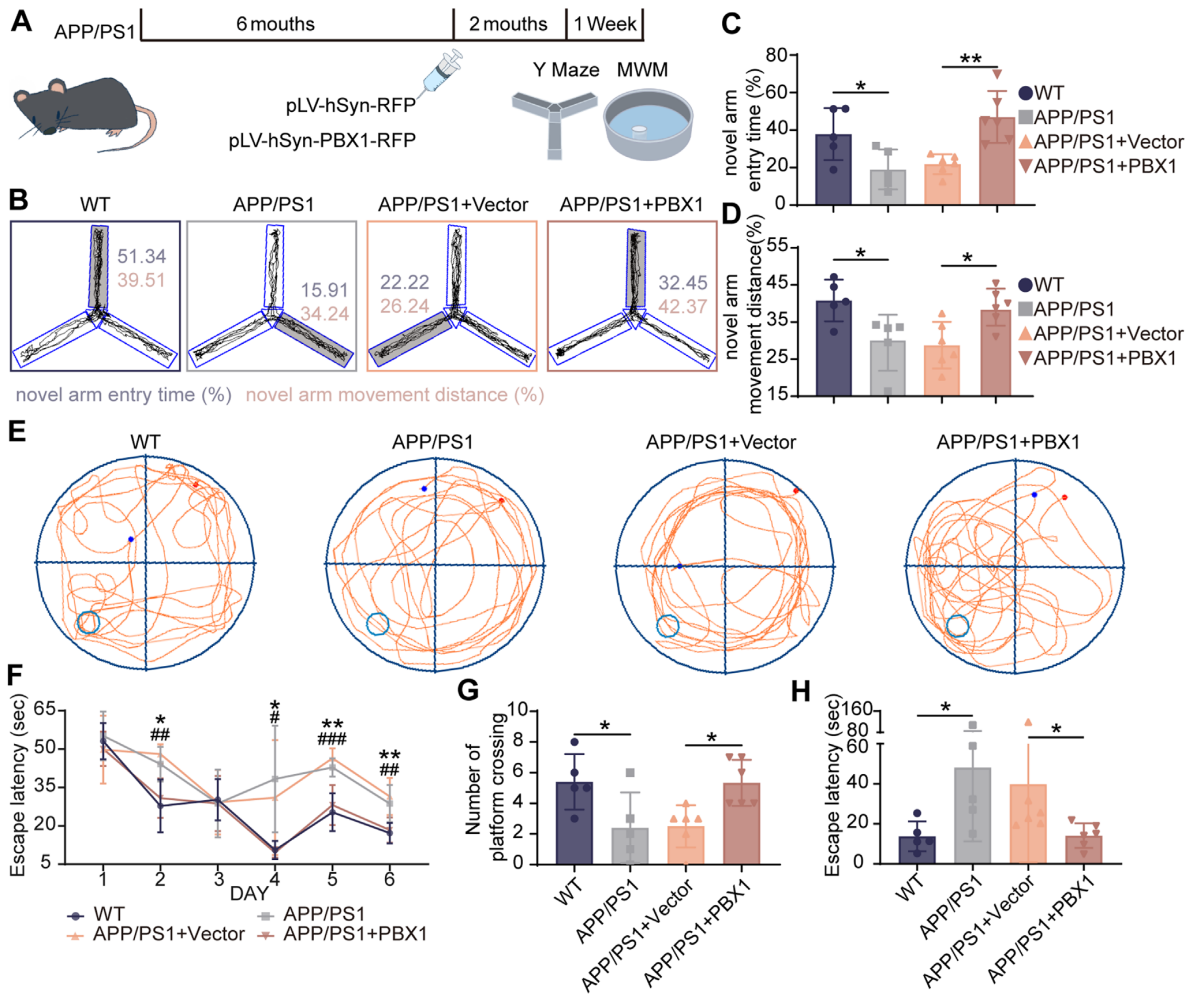
Conditional overexpression of PBX1 improved cognitive function in AD mice. (A) Experimental timeline. Six‐month‐old APP/PS1 mice were stereotaxically injected—in the hippocampal CA1 region—with lentiviral particles overexpressing a vector or PBX1. The lentiviral particle was regulated by the neuron‐specific promoter hSyn. After 2 months from injection, behavioral assessments were performed. (B) Representative Y‐maze trajectory plots depicting the novel arm (light gray) introduced during the test phase. Within each trajectory plot, blue/pink numbers indicate the novel‐arm entry time (%) and novel‐arm movement distance (%) for that animal, respectively. (C and D) Percentage of time/distance in the novel arm during the Y‐maze novel arm test. Each experimental group comprised five to six mice. (C) Kruskal–Wallis tests followed by Dunn's post hoc comparisons with Benjamini‐Hochberg correction were used for nonparametric data. (D) One‐way analysis of variance with Tukey's multiple comparison test was performed. (E) MWM swimming trajectories on Day 7 of testing. Light blue circle: Original platform position used during Days 5–6; red dot: Mouse start position at the beginning of the 60 s probe trial; blue dot: Mouse end position at trial conclusion. Group sizes: WT *n* = 5, APP/PS1 *n* = 5, APP/PS1+Vector *n* = 6, APP/PS1+PBX1 *n* = 6. (F) Escape latency over the 6 days of training; Wild‐type versus APP/PS1: *p < 0.05 and **p < 0.01; APP/PS1+Vector versus APP/PS1+PBX1: ^#^ *p* < 0.05, ^##^ *p* < 0.01, and ^###^ *p* < 0.001. Days 2, 5, and 6: One‐way analysis of variance with Tukey's multiple comparison test was performed. Day 4: Kruskal–Wallis tests followed by Dunn's post hoc comparisons with Holm correction were used for nonparametric data. (G and H) Platform crossings and initial latency to reach the target quadrant on Day 7, Kruskal–Wallis tests followed by Dunn's post hoc comparisons with Holm correction were used for nonparametric data. *p < 0.05 and **p < 0.01.

We further evaluated the therapeutic potential of PBX1 in APP/PS1 mice. PBX1‐expressing lentiviral particles were stereotaxically injected into the hippocampal CA1 region. Neuron‐specific overexpression was achieved using lentiviral particles regulated by the hSyn promoter (Figure S6). APP/PS1 mice received LV‐PBX1 or LV‐Vector injections at 6 months of age. After 2 months, behavioral assessments were performed (Figure 4A). In the Y‐maze novel arm test, LV‐PBX1‐treated APP/PS1 mice exhibited restored preference for the novel arm (Figure 4B–D). In the Morris water maze (MWM) test, LV‐PBX1‐treated APP/PS1 mice showed shorter escape latencies than APP/PS1+Vector mice (Figure 4E–H). Overall, these findings indicate that hippocampal administration of PBX1‐expressing lentiviral particles can mitigate spatial memory deficits in APP/PS1 mice.

**FIGURE 5:**
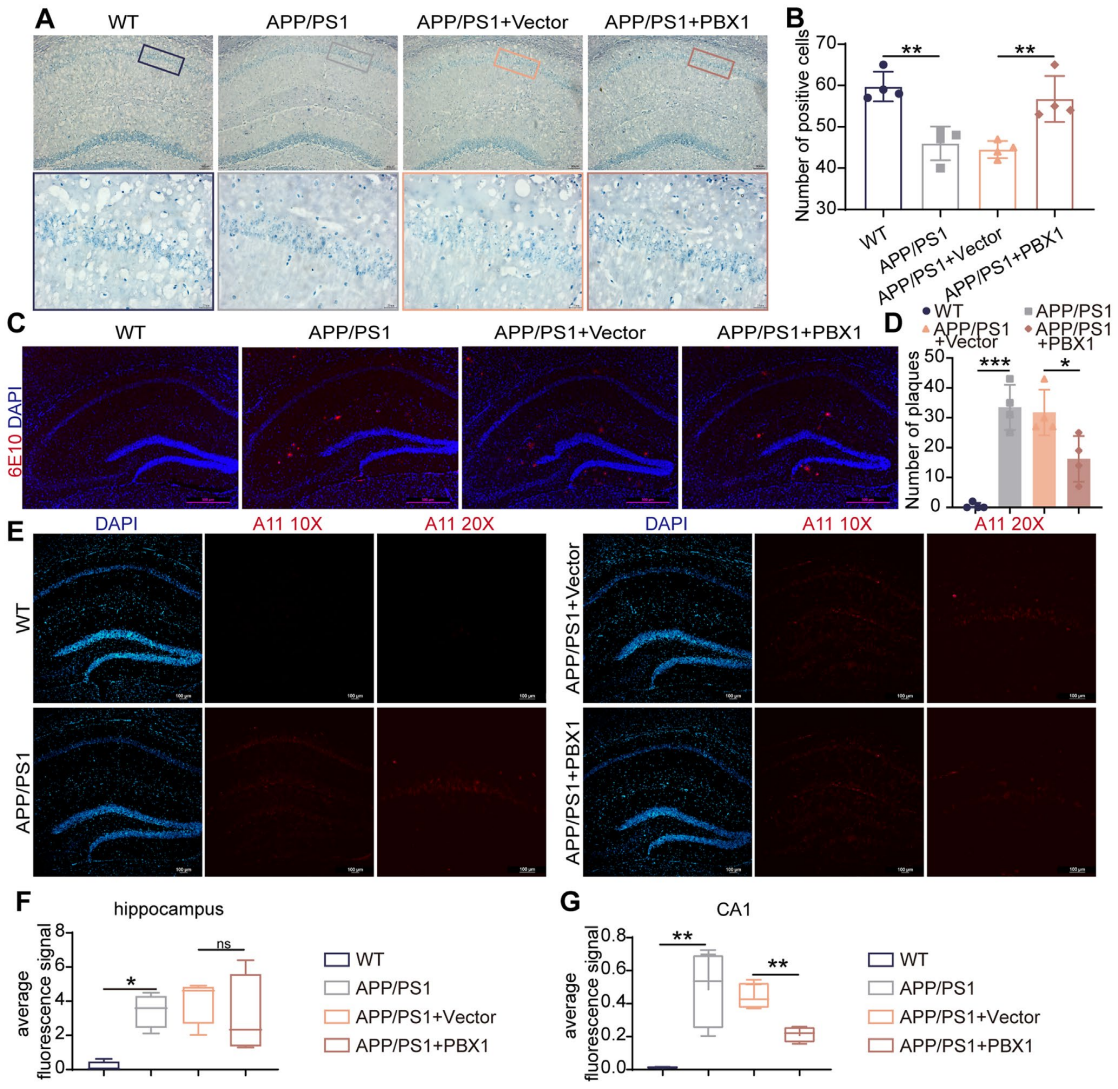
PBX1 overexpression reduced Aβ deposition in AD mice. (A) Nissl staining results for hippocampal tissues from each mouse group. (B) Quantification of Nissl‐positive neurons in the hippocampal CA1 region. Each experimental group comprised four mice. (C and D) Immunofluorescence of 6E10 (Aβ) and amount of plaques in the hippocampus. Each experimental group comprised four mice. (E–G) Immunofluorescence intensity of soluble A11 oligomers in hippocampal subregions. Each experimental group comprised four mice. Statistical analyses were performed using one‐way analysis of variance with Tukey's multiple comparison test unless otherwise specified. *p < 0.05, **p < 0.01, and ***p < 0.001.

In addition to improving cognitive function, PBX1 overexpression ameliorated AD‐associated neuropathology. Histological analysis confirmed the therapeutic benefits of PBX1. Nissl staining revealed that PBX1 overexpression improved the cytoarchitectural integrity of hippocampal CA1 neurons, as evidenced by increased density of Nissl‐positive cells and a relatively organized arrangement of Nissl bodies (*p* = 0.002, wild‐type vs. APP/PS1; *p* = 0.006, APP/PS1+Vector vs. APP/PS1+PBX1; Figure 5A,B). Notably, PBX1 reduced Aβ burden. Immunofluorescence staining indicated a 41% reduction in Aβ load (*p* = 0.006, APP/PS1+Vector vs. APP/PS1+PBX1; Figure 5C,D). This overall reduction in plaque burden occurred without a significant change in 6E10 fluorescence intensity per unit area (*p* > 0.05; Figure S7). A11 oligomer labeling confirmed selective reduction of toxic Aβ oligomers in transduced subregions (*p* = 0.003, APP/PS1+Vector vs. APP/PS1+PBX1; Figure 5E–G). Collectively, these findings suggest that hippocampal PBX1 administration not only mitigates cognitive deficits but also reduces Aβ deposition in vivo.

### 2.5 PBX1 Activates *CRTC2* Transcription to Promote the Neuroprotective CRTC2/Phosphorylated CREB Pathway

Target identification (Figure S8A) was performed to identify genes regulated by PBX1. Among the 2969 genes differentially expressed between patients with AD and individuals with normal cognitive function (GSE15222 dataset) (Webster et al. 2009), 151 overlapped with PBX1 chromatin immunoprecipitation (ChIP)‐seq targets (Zhang et al. 2020; Zheng et al. 2019). After screening, 87 neuronal genes were selected for KEGG and GO analyses (Xu et al. 2018), which revealed CRTC2 enrichment in positive regulation of CREB transcription factor activity (Figure S8B–D). During memory formation, CRTC2 interacts with phosphorylated CREB (p‐CREB) and CREB‐binding protein/p300 to form transcriptional complexes essential to CREB‐dependent gene expression (Altarejos and Montminy 2011; Hu et al. 2020; Sekeres et al. 2012).

**FIGURE 6:**
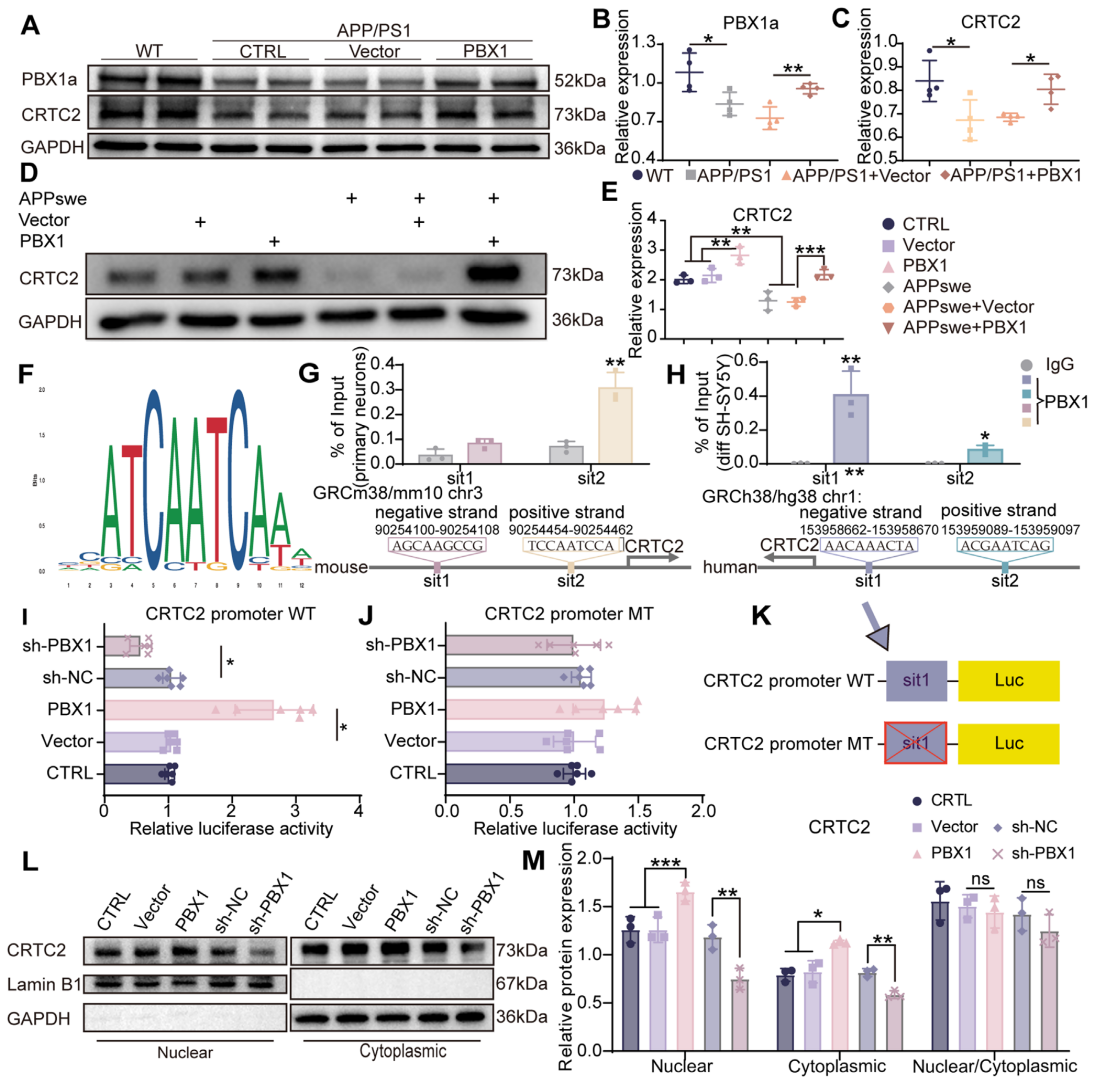
PBX1 modulated *CRTC2* promoter activity and transcription. (A–C) Levels of PBX1a and CRTC2 in the hippocampal tissues of mice across all groups, measured through Western blotting. The bar graphs depict the expression levels of these proteins to that of GAPDH. Each experimental group comprised four mice. Kruskal–Wallis tests followed by Dunn's post hoc comparisons with Benjamini–Hochberg correction were used for nonparametric data. (D and E) Level of CRTC2 in a cellular model of AD, measured through Western blotting. The scatter plot depicts the expression level of CRTC2 normalized to that of GAPDH. One‐way analysis of variance with Tukey's multiple comparison test was performed. (F) PBX1 binding motif; data were obtained from the JASPAR database. (G and H) Species‐specific ChIP–qPCR analysis of PBX1 binding at the sit1 (differentiated SH‐SY5Y cells/human) and sit2 (primary neurons/mouse) CRTC2 promoters; the independent‐samples *t‐*test was performed. (I and J) Dual‐luciferase reporter assays were performed by cotransfecting SH‐SY5Y cells that either lacked PBX1 or overexpressed it with the wild‐type or mutant CRTC2 promoter construct and the Renilla luciferase control plasmid (pRL‐TK; at a 5:1 ratio). After 48 h from transfection, the ratios of firefly luciferase activity to Renilla luciferase activity in the experimental groups were normalized to the ratio in the control group. The nonparametric Kruskal–Wallis test was performed. (K) Wild‐type and mutant CRTC2 promoter–luciferase constructs. (L) Representative Western blots showing CRTC2 distribution between cytoplasmic and nuclear fractions. Fractionation quality was validated by Lamin B1 and GAPDH. (M) Quantification of CRTC2 levels in nuclear and cytoplasmic fractions and the nuclear/cytoplasmic ratio. Protein levels were first normalized to the respective fraction‐specific loading controls and then used to compute the nuclear/cytoplasmic ratio. Statistical test: One‐way ANOVA with Tukey's multiple comparison test. *p < 0.05, **p < 0.01, and ***p < 0.001.

We verified the downregulation of hippocampal CRTC2 expression in mouse and cellular models of AD. Notably, PBX1 overexpression upregulated the expression of CRTC2 (Figure 6A–E). Bioinformatic analyses of available ChIP datasets revealed PBX1‐enriched regions within the *CRTC2* promoter. ChIP followed by qPCR confirmed the binding of PBX1 to the CRTC2 promoters in mouse hippocampal neurons, differentiated SH‐SY5Y cells and HEK293 cells (Figure 6F–H, Figure S9). Dual‐luciferase assays revealed that PBX1‐overexpressing cells exhibited 2.9 ± 0.7‐fold higher CRTC2 promoter activity than did vector control cells (*p* < 0.001). However, PBX1 knockdown reduced promoter activity to 57% ± 16% of the baseline level (*p* = 0.017). Mutation of PBX1‐binding sites in the promoter, based on ChIP–qPCR results, abolished PBX1‐dependent transactivation (Figure 6I–K). These findings suggest that PBX1 directly activates the transcription of *CRTC2* through sequence‐specific promoter interactions.

To assess effects on CRTC2 abundance and localization, we performed nuclear–cytoplasmic fractionation. PBX1 overexpression markedly increased total CRTC2, with corresponding increases in both nuclear and cytoplasmic fractions; the nuclear: cytoplasmic ratio, however, remained unchanged (Figure 6L–M). Immunoblotting for p‐CRTC2 in differentiated SH‐SY5Y cells and AD mouse models showed that PBX1 overexpression increased p‐CRTC2 levels while leaving the p‐CRTC2/CRTC2 ratio unaltered (Figure S10). Thus, PBX1 expands the total cellular pool of CRTC2, which increases the absolute amount of dephosphorylated CRTC2 available for nuclear function without changing the phosphorylation equilibrium or translocation kinetics.

Immunofluorescence corroborated these results, showing increased nuclear CRTC2 staining and enhanced colocalization of CRTC2 with p‐CREB in differentiated SH‐SY5Y cells and in the hippocampal CA1 region of APP/PS1 mice after PBX1 overexpression (Figure S11).

### 2.6 CRTC2 Is Essential for PBX1‐Mediated Neuroprotection

To confirm the role of CRTC2 in mediating the protective effect of PBX1, we knocked down *CRTC2* in a cellular AD model overexpressing PBX1. Three small interfering RNAs (siRNAs) were used for this purpose: siCRTC2‐2, siCRTC2‐268, and siCRTC2‐667. The sequences of these siRNAs are presented in Table 2. In addition, a negative control (si‐NC) was used. The experimental siRNAs significantly downregulated *CRTC2* expression at both the mRNA and protein levels (Figure S12A–C). In APPswe+PBX1 cells, *CRTC2* knockdown elevated intracellular and extracellular levels of soluble Aβ_1–42_ but not Aβ_1–40_ (Figure S12D,E). At the protein level, *CRTC2* knockdown decreased levels of both total CREB and p‐CREB (Figure S13).

To assess functional consequences on CREB activity, we performed dual‐luciferase reporter assays and found that PBX1 overexpression increased cyclic adenosine monophosphate response element (CRE)‐driven luciferase activity in APPswe+PBX1 cells relative to APPswe+Vector cells; this increase was blocked by *CRTC2* knockdown, indicating that PBX1 enhances CREB transcriptional activity via CRTC2 (Figure S12F). Furthermore, *CRTC2* knockdown mitigated the PBX1‐induced reductions in cleaved caspase‐3 and p‐Tau(S396) levels in the cellular model of AD (Figure S12G–K). Collectively, these findings indicate CRTC2 as a key mediator through which PBX1 mitigates amyloidogenesis and neurodegeneration in AD.

## 3 Discussion

PBX1 not only regulates the formation of new neurons in the subventricular zone but also supports the survival of these neurons. This protein determines the fate of early progenitor cells by promoting neuronal lineage commitment while reducing oligodendrocytic differentiation potential (Grebbin et al. 2016). PBX1 deficiency impairs neurogenesis and leads to aberrant axon guidance, immature neuronal morphology, and compromised functional maturation (Grebbin and Schulte 2017; Remesal et al. 2020; Sgadò et al. 2012; Villaescusa et al. 2016). Notably, PBX1 modulates the protein kinase B/GSK3β/CREB pathway during hippocampal neurogenesis (Wang et al. 2024) and regulates oxidative stress through NFE2L1 or FOXA1 in PD (Li et al. 2022; Villaescusa et al. 2016). Both GSK3β hyperactivation and oxidative damage are key pathogenic mechanisms in AD (Bai et al. 2022; Bian et al. 2021; Giovinazzo et al. 2021; Łuczyńska et al. 2024; Xiong et al. 2024). Genome‐wide gene expression profiling of 1647 postmortem brain tissues revealed that PBX1 is significantly and negatively correlated with both Braak stage progression and frontal lobe atrophy (Zhang et al. 2013). On the basis of evidence highlighting the downregulation of PBX1 in PD and the neuroprotective effect of PBX1 overexpression against PD models, we hypothesized that PBX1 dysregulation would be associated with AD pathogenesis.

PBX1 has multiple isoforms. Exon 7 of the PBX1 transcript is evolutionarily conserved across mammals and contains sequences essential for PBX1a expression. Skipping exon 7 results in a translational frameshift that introduces a premature termination codon in exon 8, leading to the formation of the truncated isoform PBX1b. Remesal et al. (2020) indicated that nearly all subependymal zone progenitors exclusively express PBX1b, immature migratory neuroblasts coexpress PBX1b and PBX1a, and mature dopaminergic neurons predominantly express PBX1a. Villaescusa et al. (2016) confirmed the expression of PBX1a in mature dopaminergic neurons. On the basis of these findings, we focused specifically on PBX1a in this study.

We first confirmed the downregulation of PBX1 expression in AD. Consistent downregulation of PBX1 expression was noted in hippocampal neurons from patients with AD and APP/PS1 mice as well as in cellular models (Figure 1). Restoration of PBX1 expression reduced Aβ deposition, attenuated neurodegeneration, and mitigated cognitive deficits in APP/PS1 mice (Figures 3, 4, 5). Notably, we evaluated PBX1a expression in not only patients with AD (assessed using data from the GEO database) but also cellular and mouse models of AD. Knocking down PBX1a in primary hippocampal neurons and SH‐SY5Y‐derived neuron‐like cells reduced neurite branching and impaired cell survival (Figure 2). All experiments consistently revealed substantial downregulation of PBX1 expression (Figure 1). Thus, PBX1 appears to play a regulatory role in AD pathogenesis.

Based on these findings, we hypothesized that upregulated PBX1 expression would exert neuroprotective effects against AD. Wild‐type mice and APP/PS1+LV‐hSyn‐RFP and APP/PS1+LV‐hSyn‐PBX1 transgenic mice were subjected to the Y‐maze novel arm test and the MWM test. PBX1 overexpression improved learning and memory deficits in AD models. These findings underscore the vital role of PBX1 in cognitive function.

PBX1 reduced Aβ deposition both in vivo and in vitro. In APPswe cells, PBX1 overexpression significantly decreased intracellular and extracellular soluble Aβ_1–42_ and Aβ_1–40_, while in APP/PS1 mice hippocampal PBX1 delivery reduced plaque burden and levels of soluble Aβ oligomers. In our AD cell model, the Aβ_1–42_/Aβ_1–40_ ratio was not significantly elevated, possibly reflecting model limitations. Importantly, PBX1 overexpression lowered this ratio even in non‐AD contexts (Figure S14). This shift is widely associated with reduced amyloid pathogenicity and is thereby mechanistically linked possibly to a lower AD risk (Chen and Dong 2008; Kim et al. 2007; Selkoe and Hardy 2016). Furthermore, in vitro PBX1 overexpression restored neurite length and reduced apoptosis. However, the specific downstream mediators through which PBX1 regulates neuronal morphology and function remain unclear.

Considering the regulatory role of PBX1, we performed an integrative analysis by using databases that combine PBX1 targets with genes differentially expressed between patients with AD and individuals with normal cognitive function. The results led to the hypothesis that PBX1 would exert neuroprotective effects against AD by modulating the transcriptional activity of CREB—a widely expressed transcription factor in the brain. CREB regulates genes that encode neurotransmitters, growth factors, transcription factors, signaling mediators, and metabolic enzymes, thereby serving as a central signaling hub that regulates fundamental neuronal functions—including development, plasticity, and survival—and ultimately supports higher‐order cognitive domains such as learning and memory (Grochowska et al. 2023; Kaushik et al. 2022; Kumari et al. 2023). In AD, the deposition of Aβ, upregulation of BACE1 (a rate‐limiting enzyme in the formation of Aβ) expression, and aggregation of Tau protein collectively suppress the activity of protein kinase A, thereby reducing the phosphorylation of CREB (Chen et al. 2012; Teich et al. 2015; Vitolo et al. 2002; Ye et al. 2020). Aβ‐induced disruption of extracellular signal‐regulated kinase (a member of the mitogen‐activated protein kinase subfamily) signaling (Ma et al. 2007; Zhang, Wu, et al. 2024) and abnormal activation of GSK‐3β synergistically reduce CREB‐mediated transcription (Long et al. 2015; Wu et al. 2018). CREB activity is also influenced by various cofactors. Notably, the expression of CREB‐regulated transcription coactivator 1 is downregulated in both patients and mice with AD, leading to Aβ‐induced impairments in synaptic plasticity and memory (Hu et al. 2020; Mendioroz et al. 2016; Parra‐Damas et al. 2014; Yan et al. 2021). CRTC2, similarly expressed in hippocampal CA1 neurons (Lerner et al. 2009), modulates the CREB–brain‐derived neurotrophic factor pathway (Hu et al. 2020).

We noted marked downregulation of CRTC2 both in vivo and in vitro, but PBX1 overexpression restored CRTC2 expression (Figure 6A–C). ChIP–qPCR and dual‐luciferase reporter assays indicated that PBX1 bound specific regions of the CRTC2 promoter, thereby enhancing its activity (Figure 6). Cellular investigations revealed PBX1‐dependent nuclear colocalization of CRTC2 and CREB (Figure S11). *CRTC2* knockdown diminished the effects of PBX1 on Aβ clearance, apoptosis, and CREB activity (Figure S12). These findings suggest the presence of a PBX1–CRTC2–CREB axis, the dysregulation of which leads to neurite degeneration and Aβ deposition in AD.

Previous studies reported reduced CREB mRNA after CRTC1 loss (Xia et al. 2017). Because the CRTC family comprises three members (CRTC1, CRTC2, and CRTC3) with similar modular structures (Altarejos and Montminy 2011), this provides indirect support for our observation that PBX1 upregulates CRTC2 to maintain CREB and p‐CREB levels. However, this is correlative evidence and direct functional validation of CRTC2's role is required. Loss of CRTC2 abolished PBX1's effects, supporting a PBX1–CRTC2–CREB axis that preserves CREB signaling, although whether this occurs at the transcriptional or post‐translational level remains to be determined.

This study has some limitations. Although APP/PS1 mice effectively model amyloidosis, they underrepresent Tau pathology. Future studies should investigate the activity of PBX1 in tauopathy models (e.g., PS19 mice) to evaluate its effect on phosphorylated Tau and identify PBX1/CRTC2‐regulated neuroprotective genes (e.g., genes encoding brain‐derived neurotrophic factor and synaptic proteins) through transcriptomic approaches.

## 4 Conclusion

Our study highlights PBX1 as a key transcriptional regulator that confers neuroprotection against AD through a CRTC2‐dependent CREB activation cascade. This study reframes transcriptional dysregulation as a viable therapeutic target for AD, with PBX1–CRTC2 activation representing a promising strategy for mitigating amyloidosis and neurite degeneration.

## 5 Methods

### 5.1 Plasmid and Viral Construct Generation

Three shRNAs targeting PBX1 (sh‐PBX1‐1/11/12) and one scrambled control (sh‐NC) were developed, with sh‐PBX1‐11 (5′‐TGTCCCAGCACTTGCAGGAT‐3′) exhibiting significant PBX1 knockdown efficacy. The PBX1a isoform (NM_002585.4) was inserted into pLVX‐IRES‐mCherry (YouBio, China) to create overexpression constructs, with the empty vector utilized as a control. The expression plasmid for amyloid precursor protein (APP) modeling, APPswe (Swedish mutation K670N/M671L) with puromycin resistance, together with the equivalent empty vector (pLVX‐Puro), was acquired from the Public Protein Repository in Nanjing, China. For in vivo applications, constructs for PBX1a (NM_183355.3) overexpression were developed utilizing the pLV‐hSyn‐RFP backbone vector (Addgene #22909), with the empty vector functioning as a control. The synthesized plasmids were co‐transfected with psPAX2 and pMD2.G packaging plasmids into HEK293T cells via Lipofectamine 2000 (Invitrogen). Lentiviral particles were collected from the cell supernatants and preserved at −80°C.

### 5.2 Bioinformatic Analysis of Brain Datasets

Gene expression profiles of pathologically confirmed AD patients and matched controls were obtained from the GEO database. The GSE1297 dataset (Platform GPL96) was utilized to evaluate PBX1 expression across distinct AD severity stages and age‐matched non‐demented controls. For downstream target gene identification, the GSE15222 dataset (Platform GPL570) was analyzed using the Limma R package (version 3.40.6). Differentially expressed genes (DEGs) were defined with thresholds of |log_2_FC| > 0.25 and Benjamini‐Hochberg adjusted *p* < 0.05. Potential PBX1‐regulated genes were identified by intersecting DEGs with established PBX1 target genes. Neuronally expressed genes were filtered using the AlzData database, followed by functional prioritization of candidate genes through GO and KEGG pathway enrichment analyses, with emphasis on terms associated with CREB transcriptional activation.

We also evaluated the association between PBX1 expression and AD neuropathology (Aβ load and phosphorylated Tau burden) using the CFG Rank module of AlzData (http://www.alzdata.org/); the output is shown in Figure S15.

### 5.3 Cell Culture and Transfection

SH‐SY5Y human neuroblastoma cells (Cell Bank/Stem Cell Bank of the Chinese Academy of Sciences, Serial. NO. SCSP‐5014) were grown in MEM/F12 supplemented with 10% fetal bovine serum (FBS, Gibco), 1% nonessential amino acids (NEAA, Sigma), and 1% penicillin/streptomycin. Lentiviral transduction was conducted before the initiation of differentiation. Neuronal differentiation commenced via serum reduction (1% FBS final concentration) and treatment with 10 μM all‐trans retinoic acid (RA, Sigma) under light‐protected settings (0.5 lx), with complete medium changes every 48 h for five consecutive days. Differentiated cells were subsequently cultured in neurobasal medium supplemented with 2% B‐27.

Primary hippocampal neurons were extracted from postnatal Day 0 C57BL/6J mice and cultured on poly‐L‐lysine coated 6‐well plates and 6‐cm Petri dishes. The primary neurons were cultured in neurobasal medium (Gibco) with the addition of 2% B‐27 (Gibco) in a 5% CO_2_ atmosphere at 37°C. Lentiviral constructs (PBX1, shPBX1, and empty vector controls) were introduced on Day 4, and Polybrene (Santa Cruz Biotechnology) was used as the transduction reagent. The transduction efficiency was confirmed via fluorescence microscopy at 72 h post‐infection.

### 5.4 siRNA Transfection

Three siRNAs targeting the human CRTC2 transcript (designated siCRTC2‐2, siCRTC2‐268, and siCRTC2‐667) along with a scrambled negative control siRNA (si‐NC) were synthesized by Genecfps (China), with sequences detailed in Table 1. For CRTC2 knockdown experiments, differentiated SH‐SY5Y neuron‐like cells (48 h post‐retinoic acid induction) were transfected using SuperTrans siRNA Transfection Reagent (Genecfps, Cat. No. TRS002). Briefly, for cells cultured in 6‐well plates, 75 pmol siRNA and 3.75 μL transfection reagent were mixed in OPTI‐MEM medium (Gibco) and complexed at room temperature for 20 min, then added to the cells. After 8 h of incubation, the transfection mixture was replaced with fresh medium to maintain differentiation status. Cells were harvested 72 h post‐transfection for subsequent analyses, with knockdown efficiency validated through qRT‐PCR and Western blotting. For rescue experiments in APPswe+PBX1‐overexpressing cells, siRNA transfection was performed at the aforementioned differentiation timepoint.

**TABLE 1:** The siRNA sequence of CRTC2.

Name of Oligo	Sequence (5′‐3′)	
CRTC2‐h‐268	Called sense	GCAGUUGUUUCGACUACCATT
	Antisense strands	UGGUAGUCGAAACAACUGCTT
CRTC2‐h‐2	Called sense	GGGUCUCUGCCCAAUGUUATT
	Antisense strands	UAACAUUGGGCAGAGACCCTT
CRTC2‐h‐667	Called sense	CCUGGACCCUGAAGAGACATT
	Antisense strands	UGUCUCUUCAGGGUCCAGGTT
siRNA Negative Control	Called sense	UUCUCCGAACGUGUCACGUTT
	Antisense strands	ACGUGACACGUUCGGAGAATT

### 5.5 TUNEL Staining

Apoptosis detection was carried out following the differentiation of SH‐SY5Y cells, via the YF488 TUNEL Apoptosis Kit (UElandy, China) in accordance with the manufacturer's guidelines. The percentage of TUNEL‐positive neurons (%) was calculated via Fiji ImageJ software.

### 5.6 Animals

5‐month‐old APP/PS1 transgenic mice (APPswe/PSEN1dE9) on a C57BL6/J background, along with C57BL6/J wild‐type mice, were acquired from Zhishan (Beijing) Health Medical Research Institute Co. Ltd. The human synapsin promoter (hSyn), known for its specificity to neurons, was chosen for the construction of the plasmid to facilitate the targeting of hippocampal neurons. At 6 months of age, APP/PS1 littermates were randomly assigned to three experimental groups. Two of these groups underwent bilateral stereotaxic microinjection of LV‐hSyn‐PBX1‐RFP or LV‐hSyn‐RFP viral particles into the hippocampal CA1 region, and behavioral assessments were conducted 2 months after the injections.

To reduce behavioral variation, all the subjects were male, with the exception of the pregnant C57BL/6J mice utilized for primary neuron extraction. The animals were maintained in a specific pathogen‐free environment (23°C ± 2°C, 45% ± 5% humidity, 12 h light/dark cycle) with unrestricted access to food and water.

### 5.7 Stereotaxic Injection

The mice were anesthetized through the inhalation of oxygen mixed with isoflurane at a flow rate of 1 L/min (5%[v/v] isoflurane) until the corneal and toe reflexes were no longer present. The mice were secured to the brain stereotaxic apparatus, followed by the removal of the parietal hair. Under aseptic conditions, scalp disinfection and incision preceded the creation of a stereotaxic burr hole over the CA1 hippocampal subfield using coordinates: AP −2.0 mm, ML ±1.8 mm, DV −1.8 mm. The LV‐hSyn‐PBX1‐RFP or LV‐hSyn‐RFP virus was injected bilaterally via a microsyringe. The infusion rate was set at 5 nL/s, and the needle remained in place for 10 min following the completion of the injection before being gradually withdrawn.

### 5.8 Behavioral Studies

The novel arm of the Y‐maze test and the Morris water maze (MWM) test were employed to assess cognitive impairment. The Y‐maze array was designed with three arms, and the mice were introduced to the Y‐maze for 5 min to explore freely and acclimate to the environment, which occurred 2 days prior to the experiment. In the training phase, each mouse was randomly assigned a starting arm and a novel arm. The novel arm was then closed off, and the mouse was placed in the starting arm to explore both arms for a duration of 10 min. One hour after training, the novel arm was opened, and the mice were placed in the starting arm to explore the three arms freely. The duration of time spent in each arm and the number of shuttle pathways utilized by the mice were documented via the video system. The initial 3 days of the MWM test served as the learning phase; mice were tasked with locating a submerged escape platform. Subjects failing to escape within 60 s were gently guided onto the platform and maintained there for 5 s. The platform was removed on the fourth day, and the mice were permitted to swim in the water for a duration of 60 s. During Days 5 and 6, the position of the platform was adjusted, and the training sessions were conducted four times each day. On the 7th day, the platform was removed, and the mice were permitted to swim once more for a duration of 60 s. The initial platform arrival latency and the frequency of platform crossings were documented via video recording and analytical software.

### 5.9 Immunofluorescence and Immunocytochemical Staining

After behavioral tests, mice were deeply anesthetized with an intraperitoneal injection of tribromoethanol and transcardially perfused with saline followed by 4% PFA. Brains were post‐fixed in 4% PFA (24 h), cryoprotected in 30% sucrose, embedded in OCT, and sectioned coronally at 16 μm. Sections were stored at −80°C. Cultured cells (SH‐SY5Y differentiated cells and primary hippocampal neurons) were fixed with 4% PFA (15 min) and stored at 4°C.

For the immunofluorescence experiments, the fixed sections/cells were permeabilized with 0.25% Triton X‐100 (10 min, RT), blocked with 10% goat serum (1 h, RT), and incubated with primary antibodies (4°C overnight). After PBS washes, samples were incubated with fluorophore‐conjugated secondary antibodies (1 h, RT), counterstained with DAPI, and imaged.

For the immunocytochemical staining experiments, the samples were permeabilized (1% Triton X‐100, 10 min), blocked for endogenous peroxidase (10 min), and incubated with 10% goat serum (1 h). After primary antibody incubation (4°C overnight) and PBS washes, detection was performed using the UltraSensitive SP IHC Kit (Maxim, China) with DAB chromogen. Nuclei were counterstained with hematoxylin.

The following antibodies were used: Tuj1(CST, Cat. No. #5666), Oligomer A11(Thermo Fisher Scientific, Cat. No. AHB0052), CRTC2(Proteintech, Cat. No. 12497‐1‐AP), Phospho‐Creb S133 (Huabio, Cat. No. JB25‐40), anti‐rabbit (Alexa Fluor 555, CST, Cat. No. #4413), anti‐mouse (Alexa Fluor 488, CST, Cat. No. #4408), and anti‐rabbit (Alexa Fluor 647, Abcam, Cat. No. ab150075).

### 5.10 Western Blotting

Protein concentrations of whole‐cell and tissue lysates were determined by BCA assay (Epizyme, Cat. No. ZJ102). For nuclear–cytoplasmic fractionation, proteins were extracted using the Nuclear and Cytoplasmic Protein Extraction Kit (Beyotime, Cat. No. P0027) according to the manufacturer's instructions; protein concentrations of each fraction were quantified by BCA assay. Equal amounts of protein were loaded per lane: 30 μg for 10‐well gels and 12 μg for 15‐well gels. Protein lysates denatured in loading buffer (100°C, 10 min) were subjected to SDS‐PAGE resolution and electrophoretic transfer to nitrocellulose membranes (Merck). Following a 1‐h incubation in 5% skim milk at room temperature, membranes were subsequently incubated with primary antibodies overnight at 4°C, then with HRP‐conjugated secondary antibodies for 1 h at room temperature, followed by ECL development. Band intensities were quantified by ImageJ.

The primary antibodies used were as follows: PBX1 (CST, Cat. No. #4342), caspase‐3 (Abcam, Cat. No. ab13585), cleaved caspase‐3 (CST, Cat. No. #9661), Phospho Tau S396 (Abcam, Cat. No. ab109390), APP (CST, Cat. No. #48663), CRTC2 (Proteintech, Cat. No. 12497‐1‐AP), CREB1 (Proteintech, Cat. No. 67927‐1‐Ig), Phospho‐CREB1 Ser133 (Proteintech, Cat. No. 28792‐1‐A), and Phospho‐TORC2 Ser171 (Bioss Cat. No. bs‐3415R).

### 5.11 Nissl Staining

The frozen sections were treated with Nissl staining solution at 37°C for 30 min (Biosharp, China). Neuronal degeneration in the CA1 region of the hippocampus was documented and captured via microscopy (Olympus CX33).

### 5.12 Hoechst 33342 Staining

SH‐SY5Y cholinergic neuron‐like cells, differentiated with retinoic acid, were grown in neurobasal medium enriched with B‐27. Prior to staining, the medium was removed, and Hoechst 33342 staining solution (Leagene, China) was added and incubated at 5% CO_2_ at 37°C for 20 min. The residual Hoechst 33342 solution was removed by washing with PBS, and images were acquired via a live cell workstation (Cytation5).

### 5.13 Chromatin Immunoprecipitation

Primary hippocampal neurons, differentiated SH‐SY5Y cells and HEK293T cells were crosslinked (1% formaldehyde), with chromatin and cell membrane fragmented sequentially by micrococcal nuclease (MNase, 0.4 U/μL) and ultrasonication (6 × 10 s pulses). Immunoprecipitation used the Pierce Magnetic ChIP Kit (Thermo Fisher Scientific, Cat. No. 26157). Chromatin was then immunoprecipitated by the addition of an antibody against PBX1 (Thermo Fisher Scientific, Cat. No. PA5‐17223). IgG was used as a negative control, and chromatin without an antibody was used as the input group. All the groups were subjected to crosslink reversal, and DNA purification was performed. Primers were designed in combination with the peak enriched region of the CRTC2 promoter region in the existing ChIP‐Seq of PBX1 in the Cistrome Data Browser database and analyzed by qPCR; the primer sequences are shown in Table 2.

### 5.14 Real‐Time PCR Assay

Total RNA was isolated from harvested cells using TRIzol reagent (Invitrogen). Then, we used FastKing gDNA Dispelling RT SuperMix (Tiangen) to reverse transcribe it. Subsequent RT‐qPCR analysis on a QuantStudio 5 system employed FastKing OneStep qRT‐PCR Kit (Tiangen), with primers detailed in Table 2.

**TABLE 2:** Sequences of primers used for PCR in this study.

Primer		Sequences
CRTC2	Forward	TACTTATCTCCTCCCCCAGAGTC
	Reverse	ACTTGTATGAAGGGCAGAGTCAG
CRTC2‐Human‐sit1	Forward	CCTAGCCCTGACCTGTTGC
	Reverse	AGTGGAGTCCGTTTCCTCCT
CRTC2‐Human‐sit2	Forward	AAGGGGACTATCGGGTCCTG
	Reverse	ACTGACCTGTTTTGGGACCTG
CRTC2‐Mouse‐sit1	Forward	CCTTTCCCGTTCGTAGGCTC
	Reverse	ACATCACTTCCCAGCAGTCG
CRTC2‐Mouse‐sit2	Forward	CACAGCCTCAGCGTCCAAT
	Reverse	CATGACCCCTTACCCGTGTG

### 5.15 Dual‐Luciferase Reporter Assay

A dual‐luciferase reporter experiment was employed to examine the influence of the transcription factor PBX1 on the CRTC2 promoter region. Reporter plasmids were created utilizing the pGL4.19 vector. Wild‐type plasmids were engineered by including the sit1 region from ChIP‐qPCR into the vector, whereas mutant plasmids were created by altering the predicted binding site predicted by the JASPAR database. Lipofectamine 2000‐mediated cotransfection introduced firefly luciferase reporters (wild‐type/mutant) and Renilla plasmid into PBX1 overexpressing/knockdown cells. 48 h post‐transfection, firefly luciferase activity was quantified via the Dual Luciferase Reporter Gene Assay Kit (CWBIO, Cat. No. cw9312m) and normalized against Renilla luciferase activity.

### 5.16 Amyloid‐β Detection

The levels of intracellular and extracellular soluble Aβ_1–40_ and Aβ_1–42_ were quantified via ELISA kits (Tianjin Yueteng Biotechnology, Cat. Nos. YT‐H10383 and YT‐H10178) in accordance with the manufacturer's guidelines.

### 5.17 Statistical Analysis

All the statistical analyses were conducted using SPSS Statistics 22.0 and R software. Data are presented as the mean ± standard error of the mean from a minimum of three biological replicates in the experiment. For datasets meeting assumptions of normality and homogeneity of variance, one‐way ANOVA was performed followed by Tukey's post hoc test for multiple comparisons. For nonparametric data, the Kruskal–Wallis test was conducted in SPSS. When a significant overall effect was found, post hoc pairwise comparisons were performed using Dunn's test with either Holm or Benjamini–Hochberg (BH) in R. Statistical significance was established at *p* < 0.05.

## Author Contributions

Jinyu Liu, Xiaomei Liu, and Zinan Liu conceived the study and designed the methodology. Zinan Liu wrote the original manuscript draft with support from Xiangyuan Meng in data curation and validation. Zinan Liu, Siyao Li, Yujie Wang, and Xinpeng Liu carried out experiments and performed formal analysis. Rifeng Lu and Xiaoting Meng supervised the project and administered resources. Yujie Wang conducted a thorough examination and revision of the manuscript. All authors contributed to experiments, read the final manuscript, and approved the version for publication.

## Funding

This work was supported by the National Natural Science Foundation of China (82073581 and 82273673), Jilin University (24GNYZ44, 101832020CX277 and JJKH20250213BS), and Medjaden Inc. (MJR202510105).

## Ethics Statement

All animal experimental procedures received full review and approval by the Institutional Animal Care and Use Committee (IACUC) of Jilin University. This study was conducted in accordance with the integrity policies of *Aging Cell*. No AI‐generated content was utilized in this research.

## Conflicts of Interest

The authors declare no conflicts of interest.

## Supporting information


**Figure S1:** RA‐induced differentiation of SH‐SY5Y cells into neuronal‐like cells.
**Figure S2:** PBX1 immunofluorescence intensity in hippocampal subregions.
**Figure S3:** Increased levels of cleaved caspase‐3 in primary hippocampal neurons following PBX1 knockdown.
**Figure S4:** PBX1 knockdown exerts no significant effects on neural marker expression in differentiated SH‐SY5Y cells.
**Figure S5:** PBX1 overexpression enhanced the differentiation and extended the in vitro survival of neuron‐like SH‐SY5Y cells.
**Figure S6:** Stereotaxically delivered lentiviral particle–mediated regulation of PBX1.
**Figure S7:** Quantification of 6E10 immunofluorescence intensity.
**Figure S8:** Potential downstream targets of PBX1.
**Figure S9:** PBX1 Binding to the *CRTC2* Promoter.
**Figure S10:** CRTC2 and CREB1 Western blot analysis.
**Figure S11:** PBX1 modulated the expression of CRTC2 and its colocalization with p‐CREB.
**Figure S12:** CRTC2 knockdown diminished PBX1‐mediated neuroprotection in vitro.
**Figure S13:** Western blot analysis of CRTC2 and CREB1 signaling in APPswe cells under various perturbations.
**Figure S14:** Aβ_1–42_/Aβ_1–40_ ratio in extracellular and intracellular fractions.
**Figure S15:** PBX1 correlation with AD pathology from AlzData CFG Rank analysis.

## Data Availability

This study utilized publicly available datasets from the following sources: Gene expression profiles of brain tissues from Alzheimer's disease patients and neuropathologically normal controls were acquired from the NCBI GEO database (https://www.ncbi.nlm.nih.gov/geo/) via accession numbers GSE1297 and GSE15222. PBX1 target gene binding sites were obtained from Cistrome DB (http://cistrome.org/db/#/, Dataset ID: 67939) and hTFtarget (https://guolab.wchscu.cn/hTFtarget/#!/). Neuronal gene expression annotations were derived from AlzData (http://www.alzdata.org/), an integrated multi‐omics platform for Alzheimer's disease. All primary datasets are accessible through their respective portals. Data supporting the findings of this study are available from the corresponding author upon reasonable request.
