# Supplementary material for: PBX1 Improves Cognition and Reduces Amyloid‐β Pathology in APP/PS1 Mice by Transcriptionally Activating the CRTC2–CREB Pathway
Source: Aging Cell. 2025 Dec 4;25(1):e70311. doi: 10.1111/acel.70311 (PMC12741244; doi:10.1111/acel.70311)
Supplement: Supplementary file 1 — Figure S1: RA‐induced differentiation of SH‐SY5Y cells into neuronal‐like cells. Figure S2: PBX1 immunofluorescence intensity in hippocampal subregions. Figure S3: Increased levels of cleaved caspase‐3 in primary hippocampal neurons following PBX1 knockdown. Figure S4: PBX1 knockdown exerts no significant effects on neural marker expression in differentiated SH‐SY5Y cells. Figure S5: PBX1 overexpression enhanced the differentiation and extended the in vitro survival of neuron‐like SH‐SY5Y cells. Figure S6: Stereotaxically delivered lentiviral particle–mediated regulation of PBX1. Figure S7: Quantification of 6E10 immunofluorescence intensity. Figure S8: Potential downstream targets of PBX1. Figure S9: PBX1 Binding to the CRTC2 Promoter. Figure S10: CRTC2 and CREB1 Western blot analysis. Figure S11: PBX1 modulated the expression of CRTC2 and its colocalization with p‐CREB. Figure S12: CRTC2 knockdown diminished PBX1‐mediated neuroprotection in vitro. Figure S13: Western blot analysis of CRTC2 and CREB1 signaling in APPswe cells under various perturbations. Figure S14: Aβ1–42/Aβ1–40 ratio in extracellular and intracellular fractions. Figure S15: PBX1 correlation with AD pathology from AlzData CFG Rank analysis. [file ACEL-25-e70311-s001.docx]

**Supplementary Figures**

**Abbreviation:** RA, retinoic acid; FBS, fetal bovine serum; ChAT, choline acetyltransferase; AD, Alzheimer’s disease.


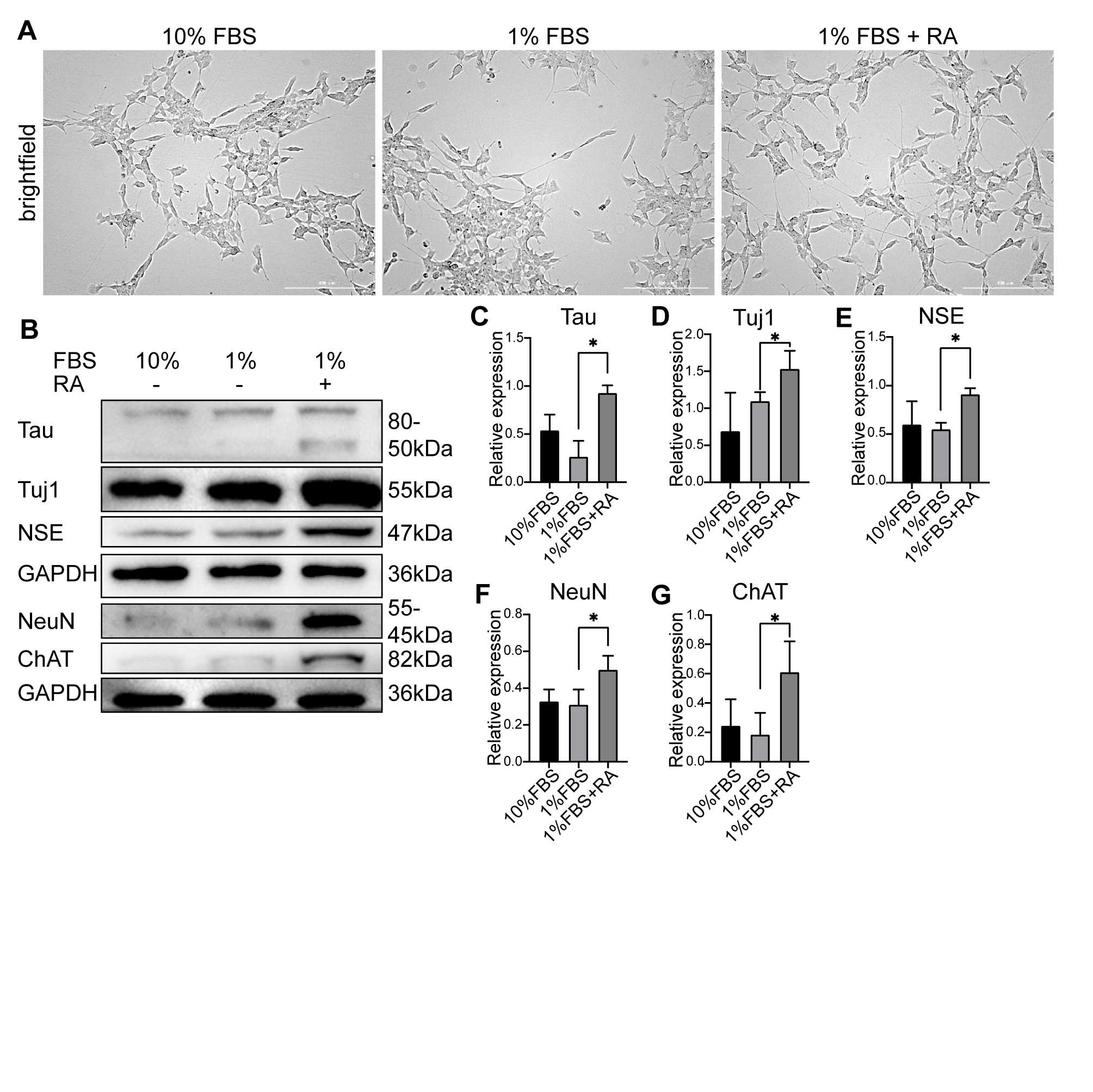


**Supplementary Figure S1. RA-induced differentiation of SH-SY5Y cells into neuronal-like cells.**

(A) Bright-field microscopy indicating increased neurite outgrowth and elongation in RA-treated cells.

(B–G) Levels of various neuronal markers (Tau, NeuN, Tuj1, and NSE) and a cholinergic marker (ChAT), accompanied by histograms presenting statistical results.

**P* < 0.05.


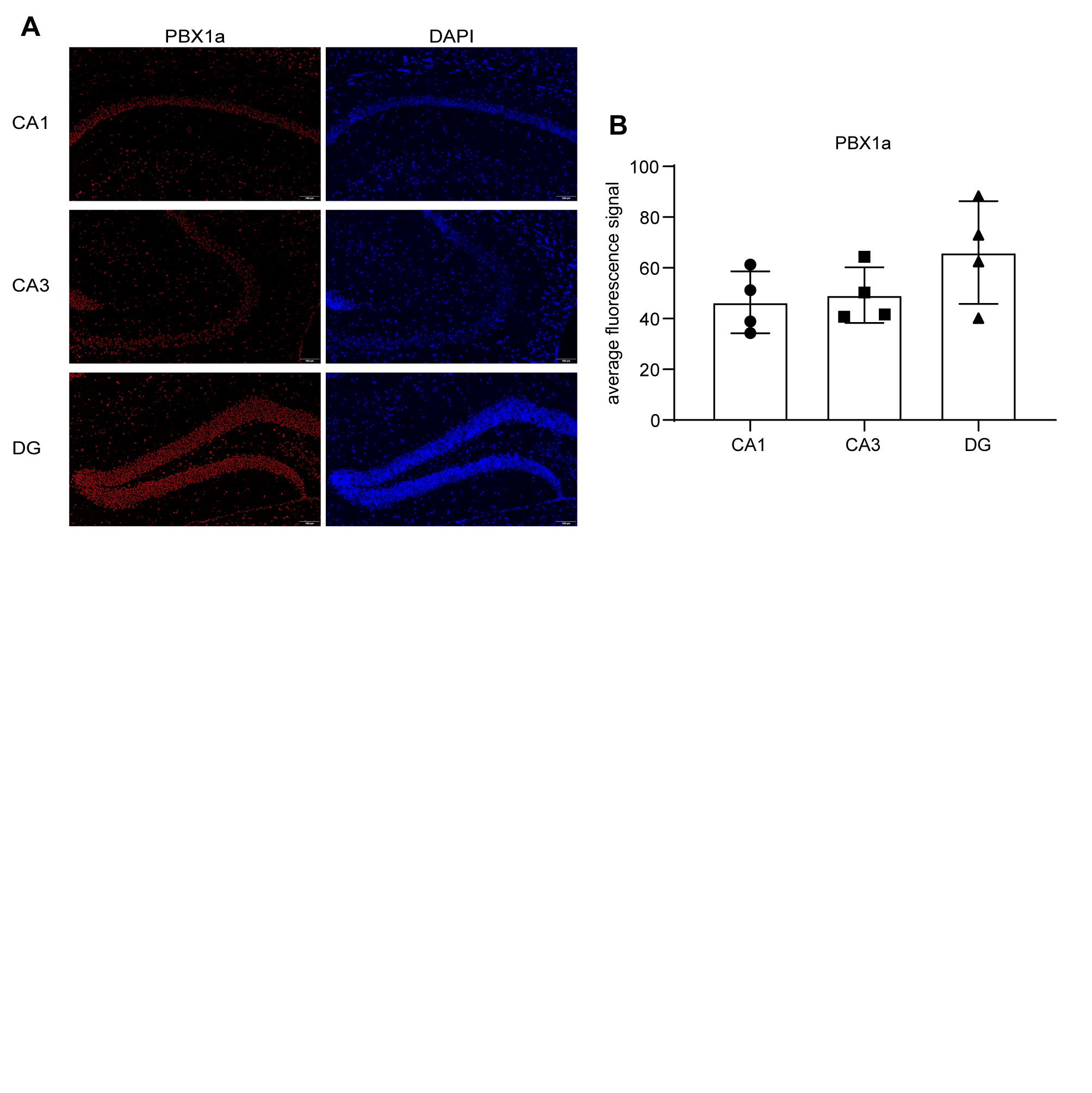


**Supplementary Figure S2. PBX1 immunofluorescence intensity in hippocampal subregions.**

(A) Representative images of PBX1 immunofluorescence (red) and DAPI (blue) in hippocampal CA1, CA3, and DG regions, acquired using a fluorescence microscope.

(B) Quantification of mean PBX1 fluorescence intensity in CA1, CA3, and DG. Statistical analysis by one-way ANOVA showed no significant difference among the groups (*P* > 0.05).


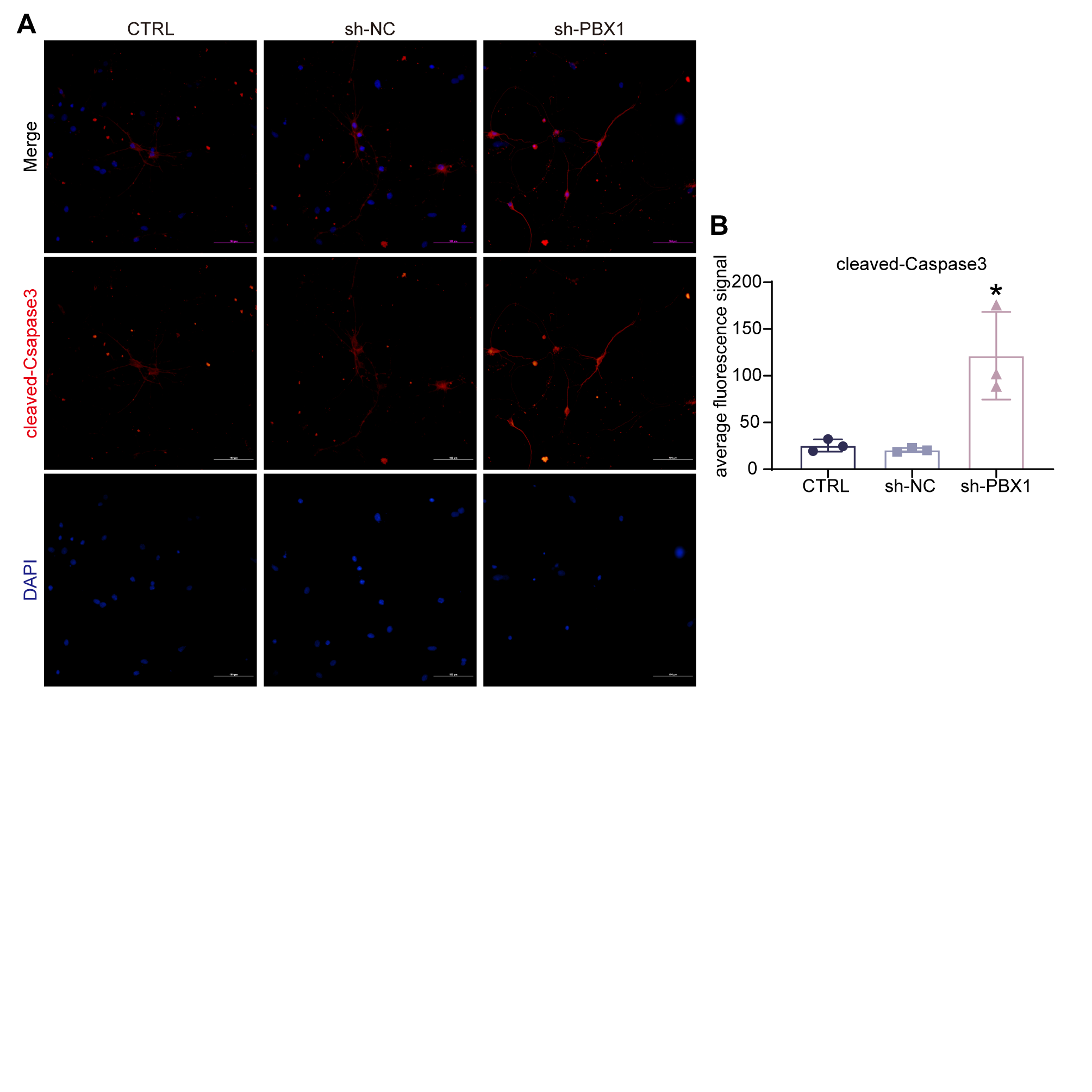


**Supplementary Figure S3. Increased levels of cleaved caspase-3 in primary hippocampal neurons following PBX1 knockdown.**

(A) Immunofluorescence of cleaved caspase-3.

(B) Bar graphs depicting the mean fluorescence intensity of cleaved caspase-3.

**P* < 0.05.

**
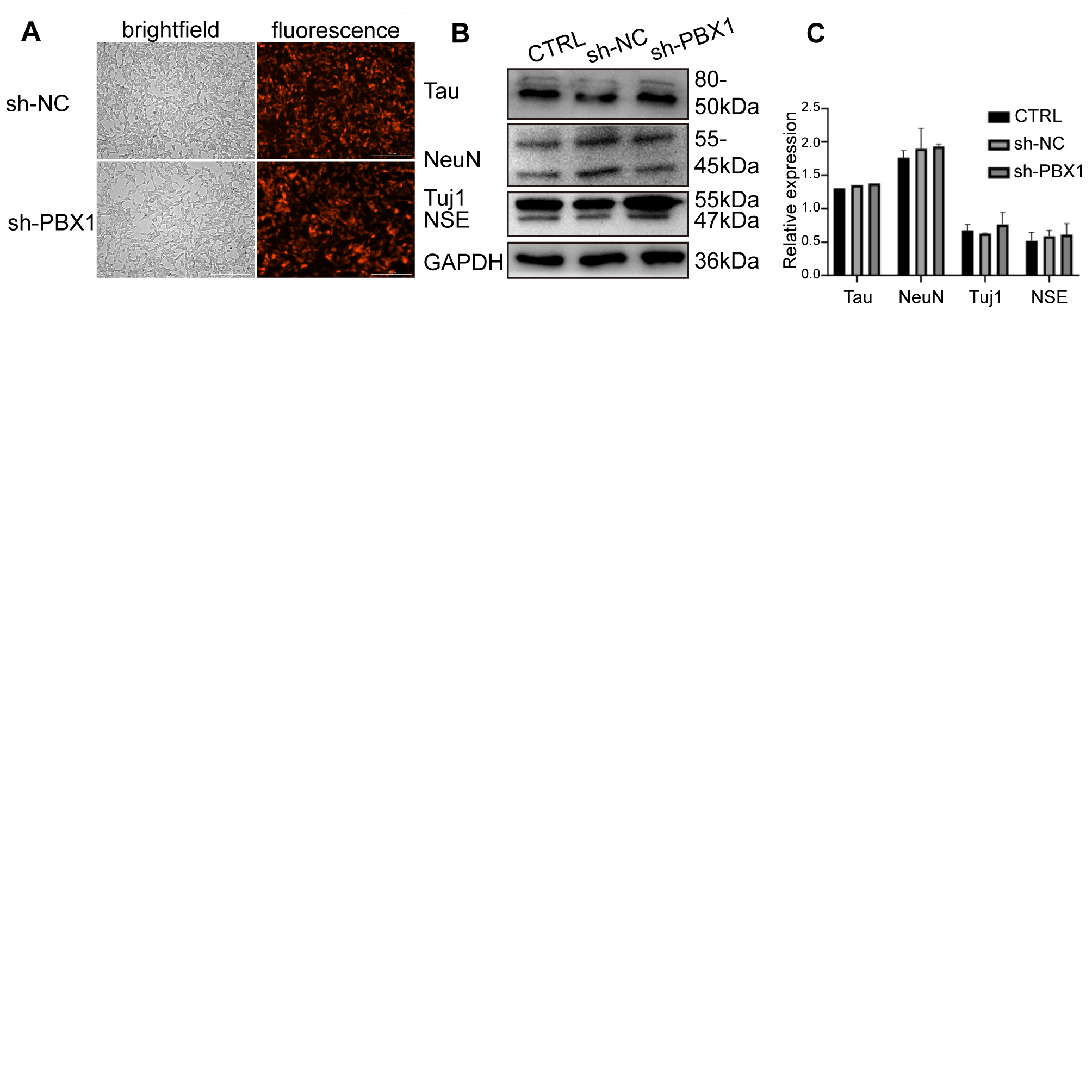
**

**Supplementary Figure S4. PBX1 knockdown exerts no significant effects on neural marker expression in differentiated SH-SY5Y cells.**

(A) Efficiency of lentiviral transduction, evaluated through fluorescence microscopy.

(B) Levels of Tau, NeuN, Tuj1, and NSE in differentiated SH-SY5Y cells lacking PBX1, measured through Western blotting.

(C) Statistical analysis of Western blotting results indicated no significant between-group differences.


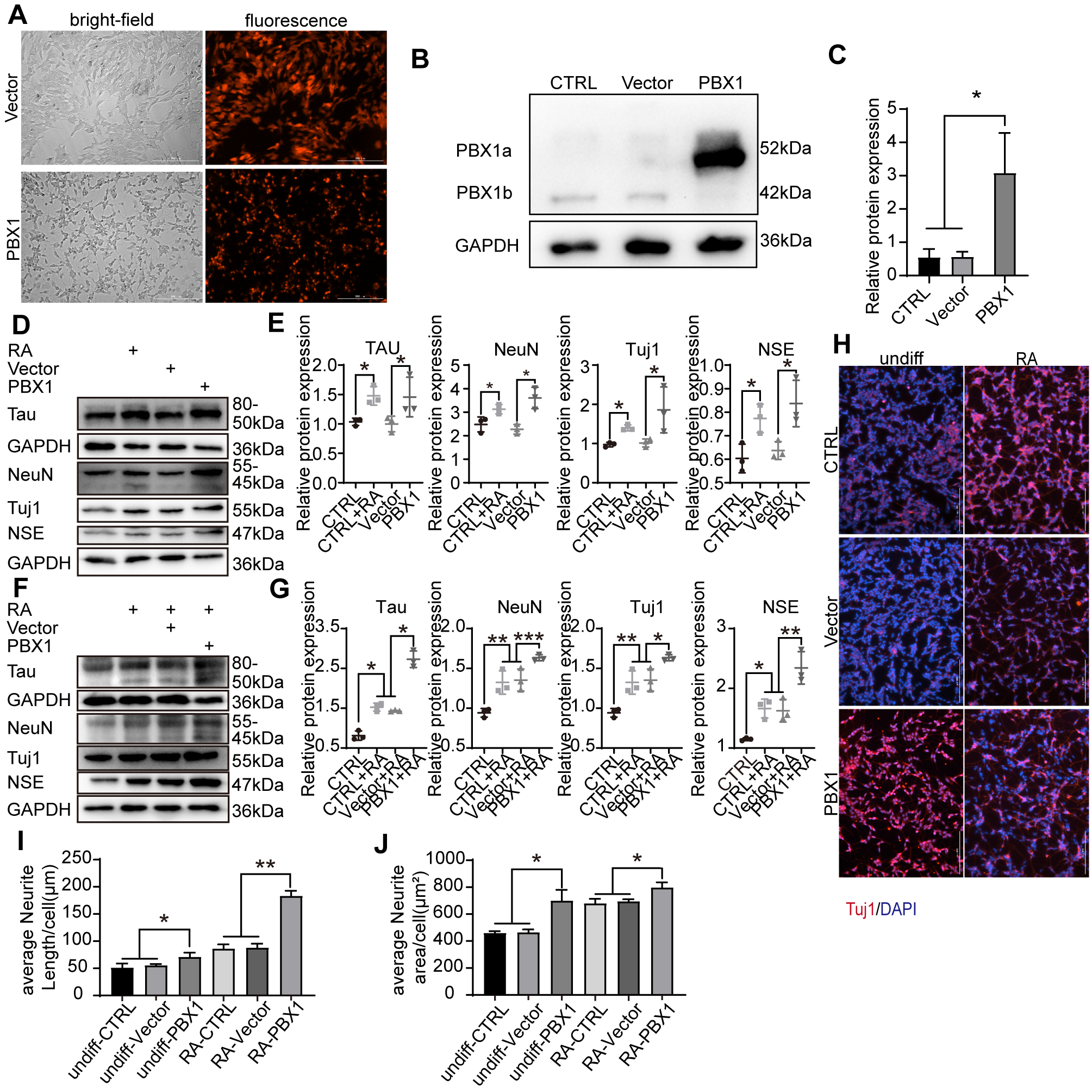


**Supplementary Figure S5. PBX1 overexpression enhanced the differentiation and extended the in vitro survival of neuron-like SH-SY5Y cells.**

(A) Efficiency of lentiviral transduction, evaluated through fluorescence microscopy.

(B and C) PBX1 overexpression confirmed through Western blotting; the histograms present quantitative results.

(D and E) Levels of various neuronal markers (Tau, NeuN, Tuj1, and NSE) in PBX1-overexpressing cells not subjected to RA treatment and those treated with RA (positive control), measured through Western blotting.

(F and G) Levels of various neuronal markers in RA-treated vector control and PBX1-overexpressing cells, measured through Western blotting.

(H–J) Tau immunofluorescence labeling with neurite morphometrics, analyzed using the Neuron J plugin in ImageJ. The results indicated measurements of neurite length and density.

**P* < 0.05 and ***P* < 0.01.

**
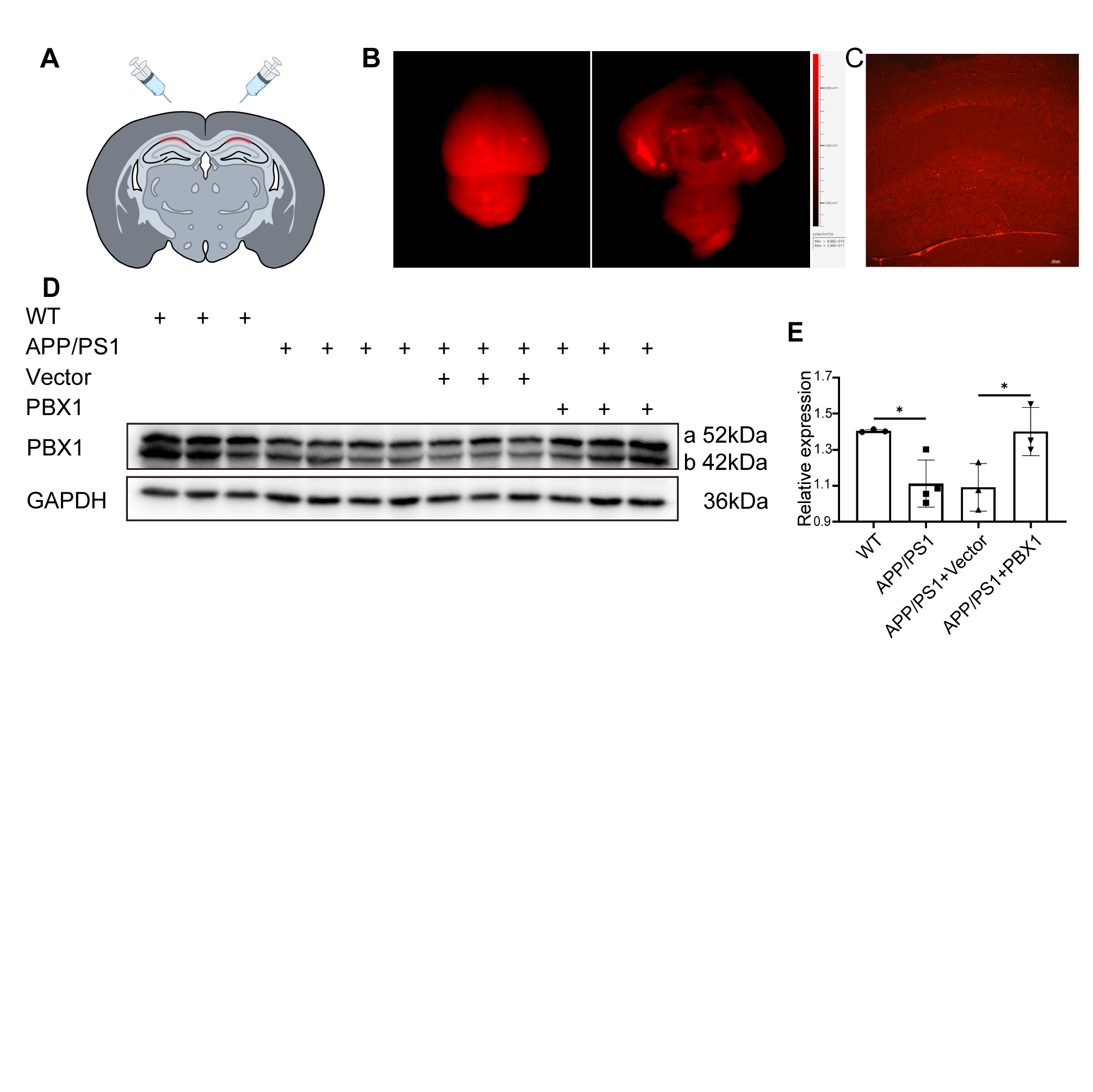
**

**Supplementary Figure S6. Stereotaxically delivered lentiviral particle–mediated regulation of PBX1.**

(A) Diagram depicting stereotaxic injection into the mouse hippocampus.

(B) In vivo fluorescence imaging confirming successful transduction of lentiviral particles.

(C) Regional transduction selectivity in the CA1 pyramidal layer, confirmed through epifluorescence imaging.

(D and E) Level of PBX1 in the mouse hippocampus, measured through Western blotting. The bar graph depicts the expression level of PBX1a normalized to that of

GAPDH.

**P* < 0.05.

**
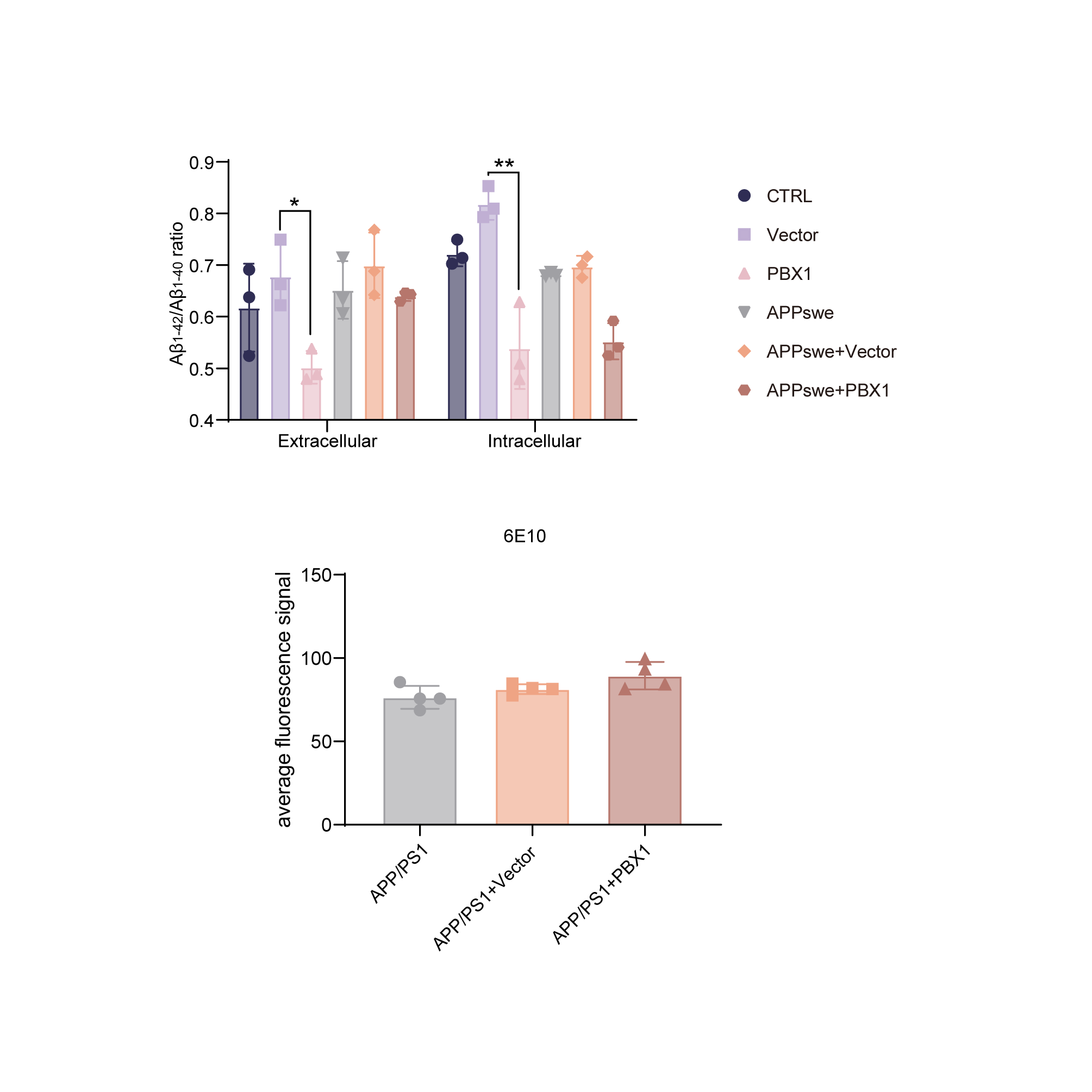
**

**Supplementary Figure S7. Quantification of 6E10 immunofluorescence intensity.**

Bar graph showing average 6E10 fluorescence intensity measured in brain sections from APP/PS1, APP/PS1+Vector and APP/PS1+PBX1 groups. Statistical analysis by one-way ANOVA showed no significant difference among the groups (*P* > 0.05).


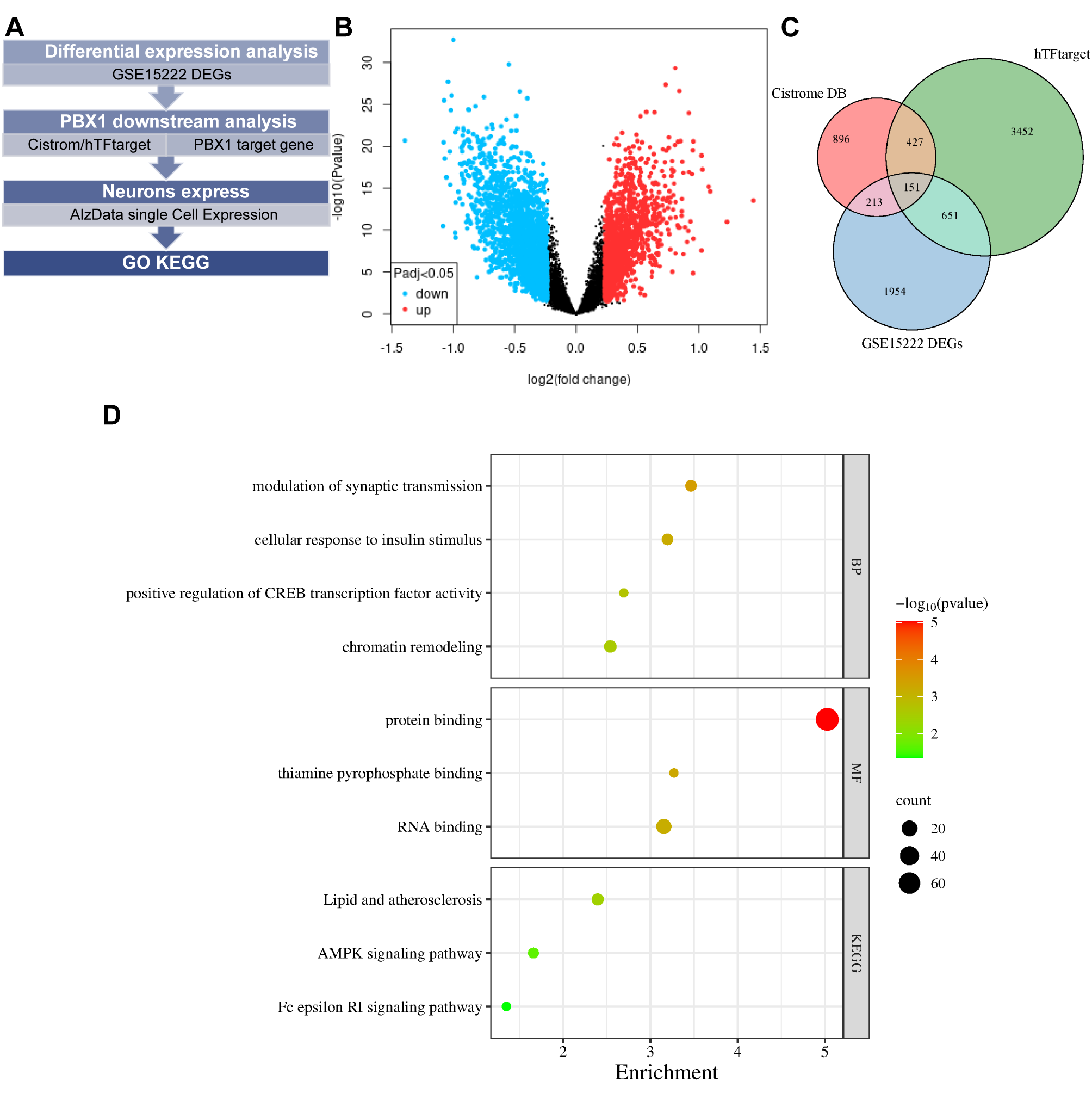


**Supplementary Figure S8. Potential downstream targets of PBX1.**

The potential downstream targets of PBX1 were investigated using data from the GSE15222 dataset of the Gene Expression Omnibus database.

(A) Flowchart depicting the process of dataset analysis.

(B) Analysis of differentially expressed genes between patients with AD and individuals with normal cognitive function. Analysis of transcriptomic data revealed differentially expressed genes associated with AD (|logFC| > 0.25; false discovery rate < 0.05).

(C) Overlapping between differentially expressed genes and PBX1 targets was investigated. A total of 151 consensus candidates were identified from a Venn diagram intersecting PBX1 ChIP-seq targets with genes dysregulated in AD.

(D) Functional enrichment analysis was performed using the DAVID tool, with results from GO and KEGG analyses.


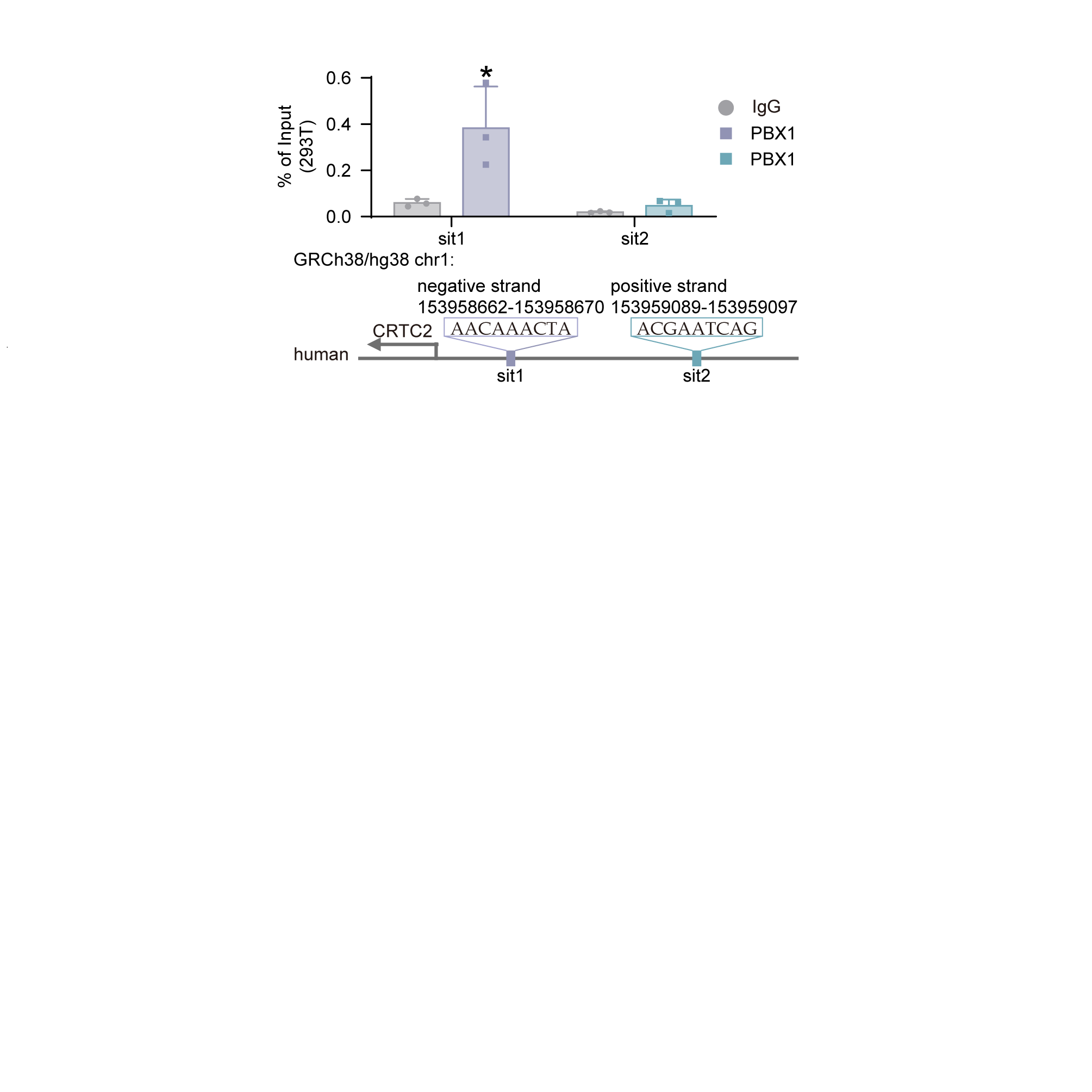


**Supplementary Figure S9. PBX1 Binding to the *CRTC2* Promoter.**

ChIP–qPCR analysis in HEK293T cells confirmed the binding of PBX1 to the human *CRTC2* promoter at site 1 (sit1). Statistical significance was determined by an independent-samples t-test.

**P* < 0.05


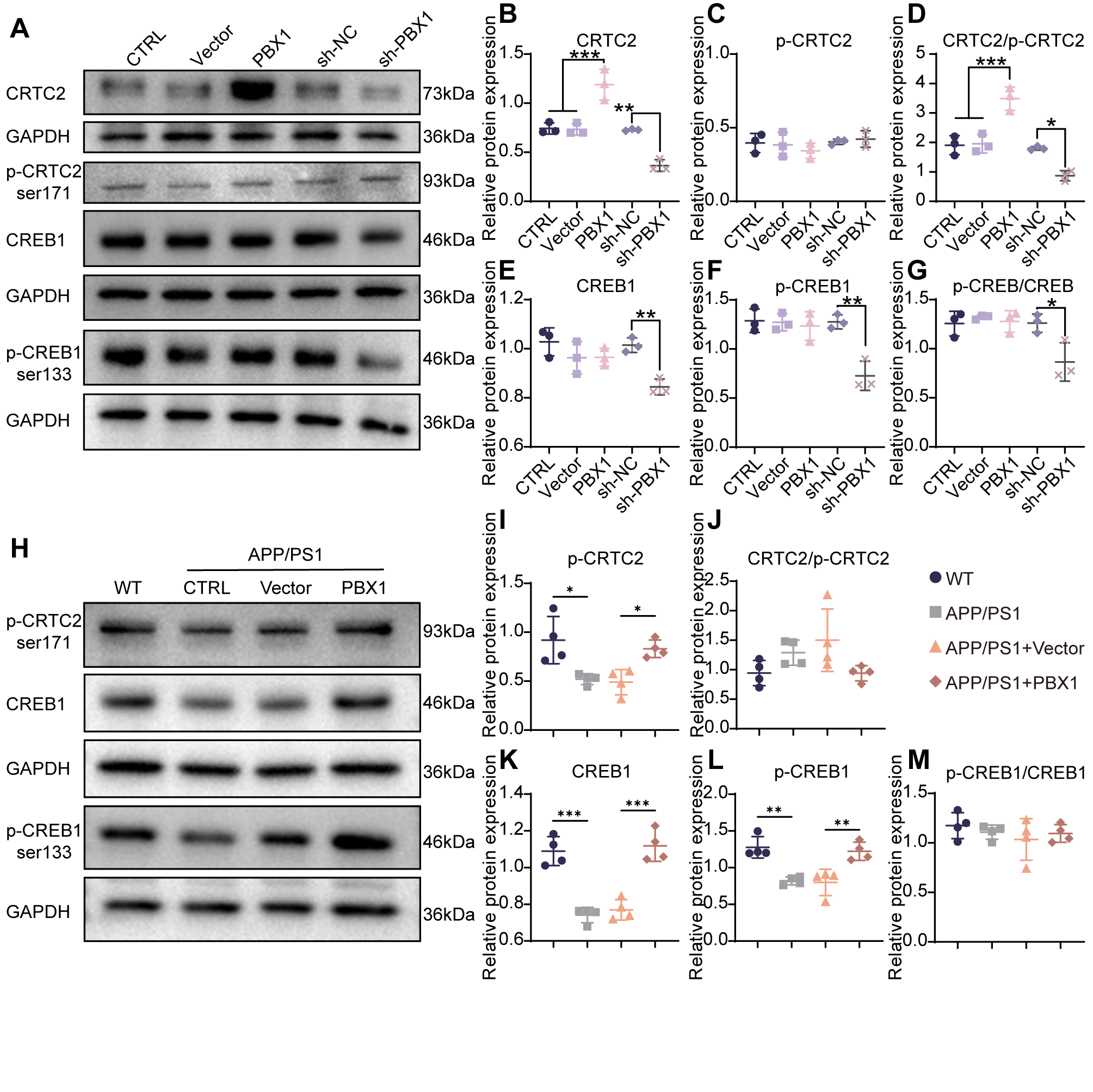


**Supplementary Figure S10. CRTC2 and CREB1 Western blot analysis.**

(A) Representative Western blots for CTRL, Vector, PBX1, sh‑NC and sh‑PBX1 groups showing CRTC2, p‑CRTC2, CREB1 and p‑CREB1 bands.

(B–G) Quantification of CRTC2, p-CRTC2, p-CRTC2/CRTC2 ratio, CREB1, p-CREB1and p-CREB1/CREB1 ratio in cell lines, normalized to GAPDH. Statistical tests: one‑way ANOVA with Tukey’s multiple comparisons.

(H) Representative immunoblots of p-CRTC2, CRTC2, CREB1, and p-CREB1 in hippocampal tissues from WT, APP/PS1, APP/PS1+Vector, and APP/PS1+PBX1 mice.

(I–M) Quantification of p-CRTC2, p-CRTC2/CRTC2 ratio, CREB1, p-CREB1 and p-CREB1/CREB1 ratio in mouse tissues, normalized to GAPDH. Statistical tests: one‑way ANOVA with Tukey’s multiple comparisons.

**P* < 0.05, ***P* < 0.01, ****P* < 0.0001.

**
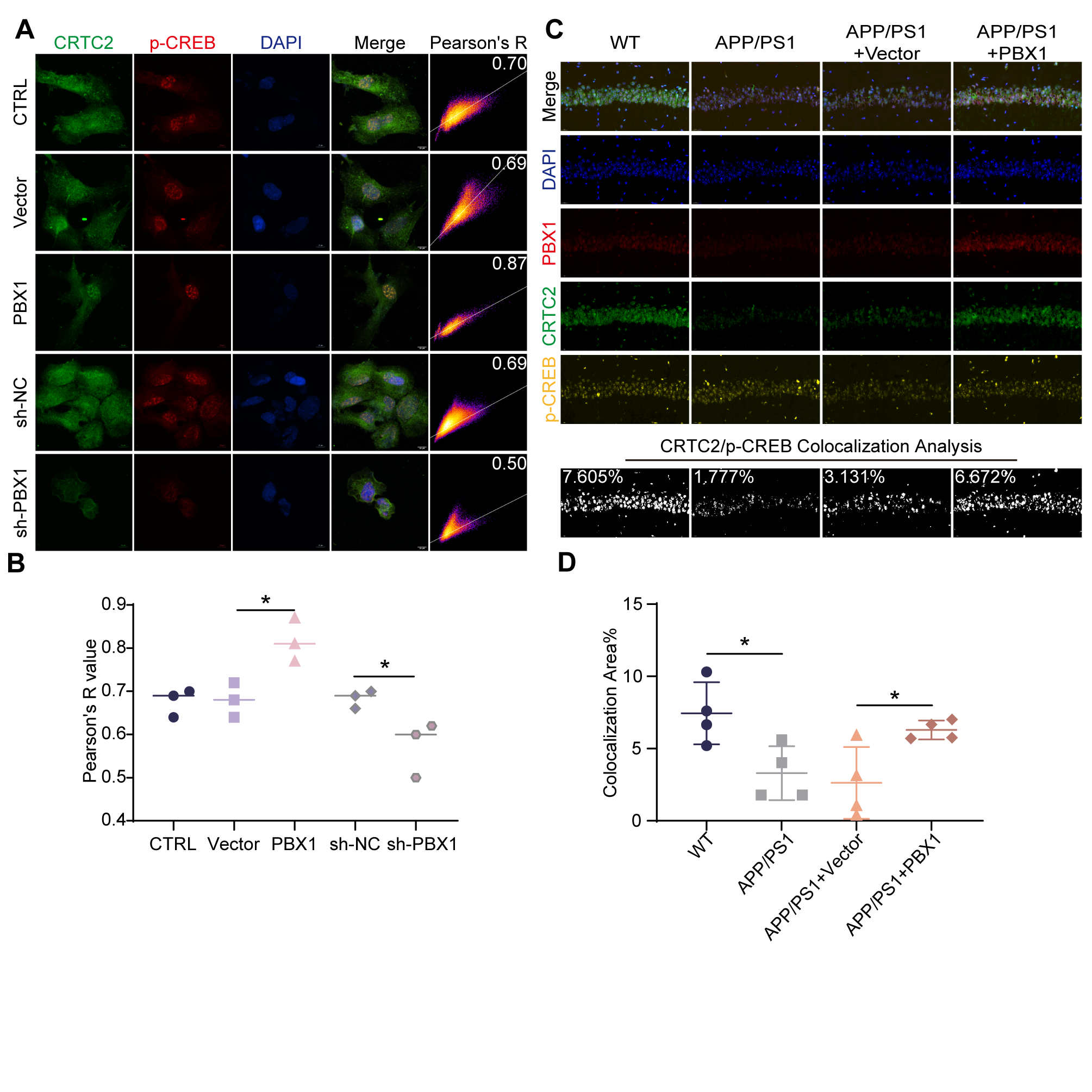
**

**Supplementary Figure S11. PBX1 modulated the expression of CRTC2 and its colocalization with p-CREB.**

(A and B) Immunofluorescence of CRTC2 and p-CREB from differentiated SH-SY5Y cells lacking PBX1 or overexpressing it and vector control cells. Colocalization analysis was performed using the Colocalization Finder plugin in ImageJ. The scatter plot presents Pearson correlation coefficients. (C and D) Results of triple immunofluorescence staining of brain tissues from experimental mice: PBX1, CRTC2, and p-CREB were targeted. Areas with CRTC2–p-CREB colocalization were quantified. The scatter plot depicts the percentage of the total area occupied by colocalized signals. For clarity, the p‑CREB immunofluorescence images in panel 7C were contrast‑adjusted globally in ImageJ using identical Minimum and Maximum display values across all groups. Quantification was performed on the original, unadjusted images. One-way analysis of variance with Dunnett’s multiple comparison test was performed. **P* < 0.05.

**
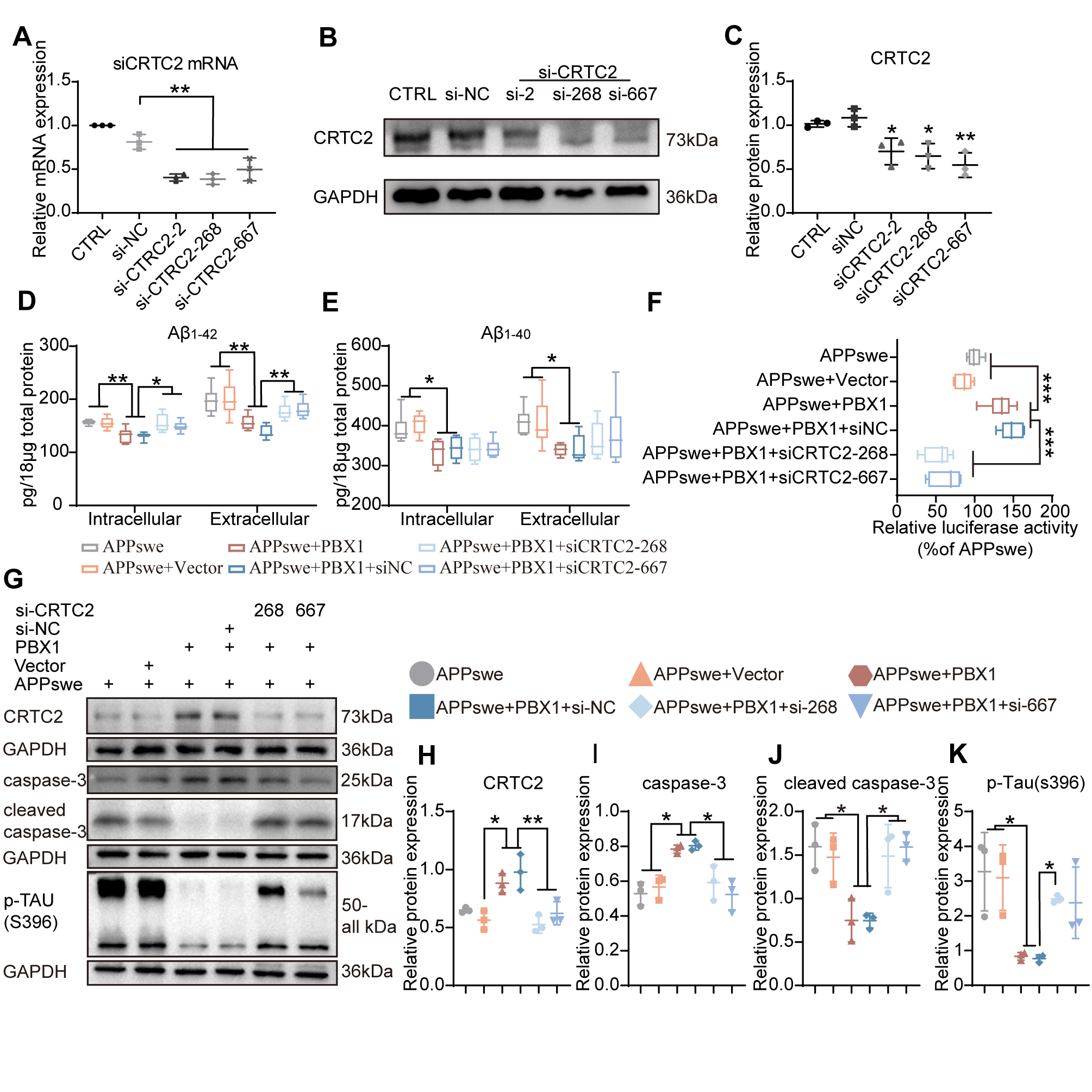
**

**Supplementary Figure S12. *CRTC2* knockdown diminished PBX1-mediated neuroprotection in vitro.**

(A) CRTC2 mRNA levels in SH-SY5Y cells transfected with siCRTC2-2, siCRTC2-267, siCRTC2-667, or si-NC. (B, C) Level of CRTC2 in each *CRTC2* knockdown group, measured through Western blotting. The scatter plot depicts the expression level of CRTC2 normalized to that of GAPDH. (D and E) Intracellular and extracellular levels of soluble Aβ_1–42_ and Aβ_1–40_ in each group, measured through an enzyme-linked immunosorbent assay. (F) Dual-luciferase reporter assays were performed by cotransfecting SH-SY5Y cells with a CREB-responsive firefly luciferase reporter construct (pGL4.19-TA-CRE) along with the Renilla luciferase control plasmid (pRL-TK) at a 5:1 ratio. Luciferase activity was measured at 48 h after transfection. The ratios of firefly luciferase activity to Renilla luciferase activity in the experimental groups were normalized to the ratio in the control group (transfected with APPswe). (G) Bands of CRTC2, caspase-3, cleaved caspase-3, and p-Tau(S396) after Western blotting. (H–K) Protein expression ratios. K: the nonparametric Kruskal–Wallis test was performed. Statistical analyses were performed using one-way analysis of variance with Tukey’s multiple comparison test unless otherwise specified. **P* < 0.05, ***P* < 0.01, and ****P* < 0.001.


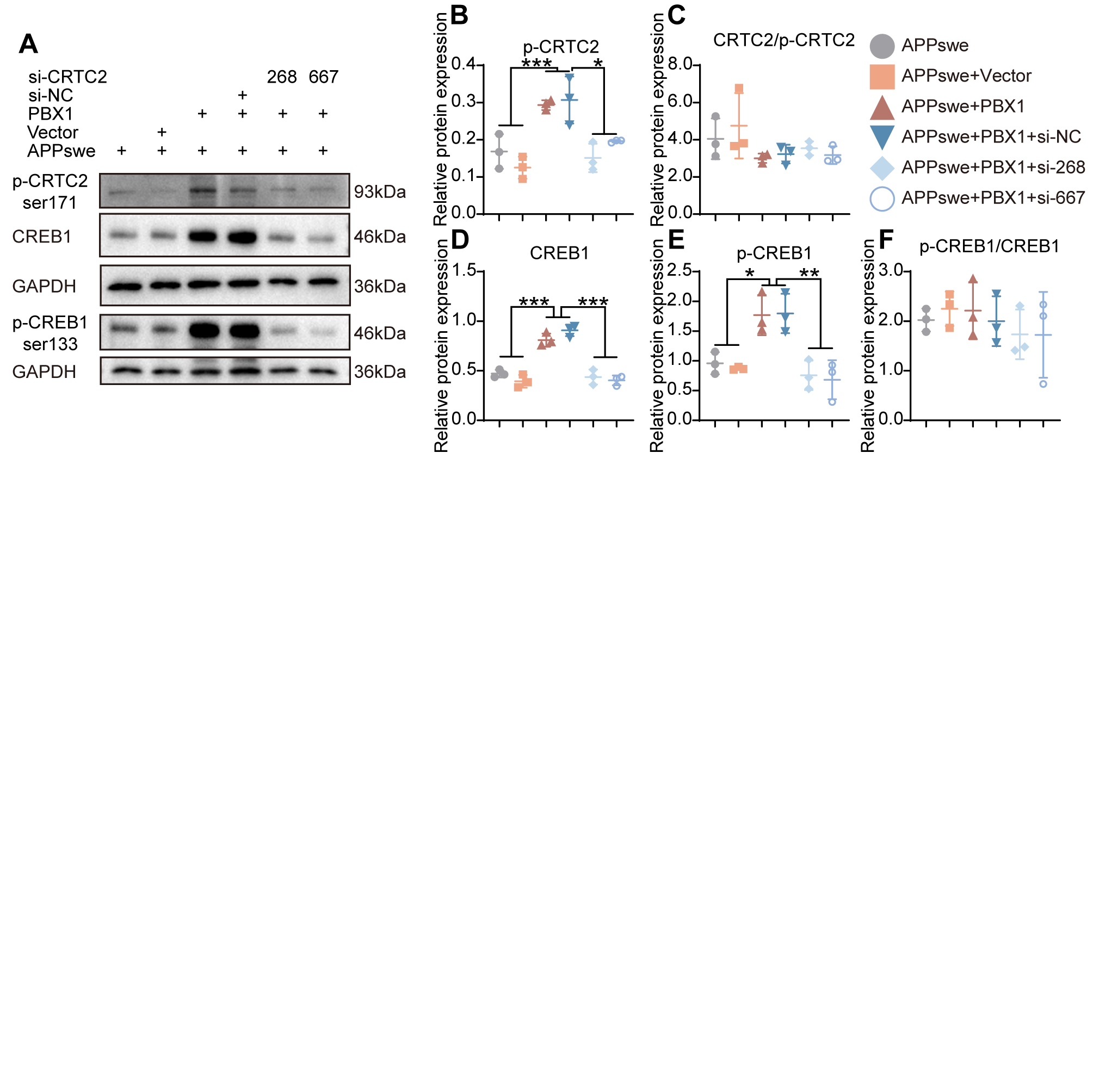


**Supplementary Figure S13.** **Western blot analysis of CRTC2 and CREB1 signaling in APPswe cells under various perturbations.**

(A) Representative immunoblots of p‑CRTC2, CREB1, p‑CREB1 and GAPDH in cells under the following conditions: APPswe, APPswe +Vector, APPswe+PBX1, and APPswe+PBX1 with si‑NC, si‑CRTC2‑268, or si‑CRTC2‑667.

(B–F) Quantification of p‑CRTC2, p‑CRTC2/CRTC2 ratio, CREB1, p‑CREB1 and p‑CREB1/CREB1 ratio. All protein levels were normalized to GAPDH. Statistical tests: one‑way ANOVA with Tukey’s multiple comparisons.

**P* < 0.05, ***P* < 0.01, ****P* < 0.0001.

**
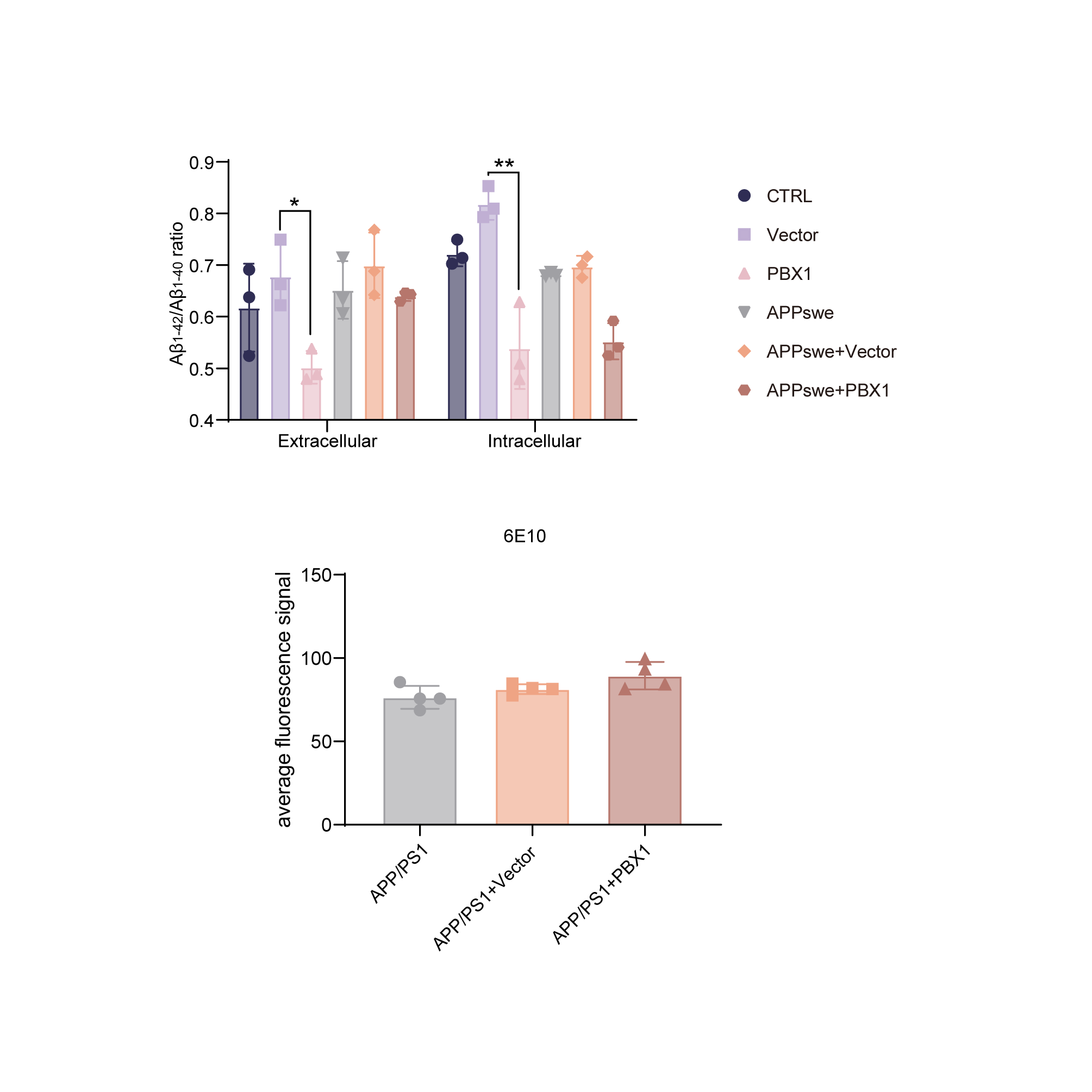
**

**Supplementary Figure S14. Aβ_1–42_/Aβ_1–40_ ratio in extracellular and intracellular fractions.**

Bar graph showing the Aβ1–42/Aβ1–40 ratio measured in extracellular and intracellular fractions. One-way ANOVA with Tukey’s post hoc test.

**P* < 0.05, ***P* < 0.01.


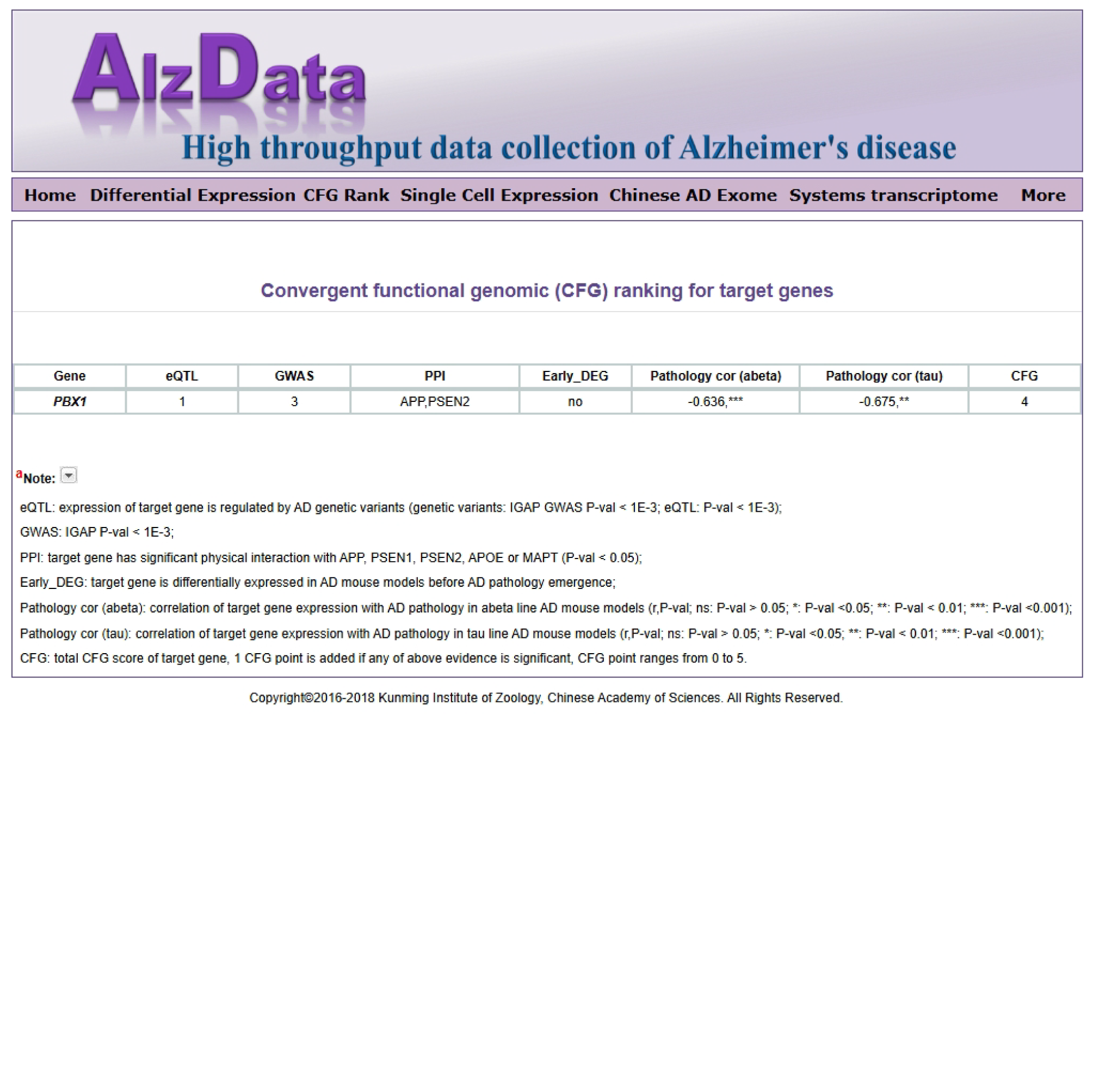


**Supplementary Figure S15. PBX1 correlation with AD pathology from AlzData CFG Rank analysis.**

Result generated by the AlzData CFG Rank module demonstrate a significant negative correlation between PBX1 expression and the severity of Aβ pathology (r = -0.636, ****P* < 0.001) and Tau pathology (r= -0.675, ***P* < 0.01) in AD mouse models.
